# Corynebacteria from the respiratory microbiota modulate inflammatory responses and associate with a reduced pneumococcal burden in the lungs

**DOI:** 10.3389/fcimb.2024.1530178

**Published:** 2025-01-28

**Authors:** Caroline Bergenfelz, Phuong Do, Liv Larsson, Hanna Ivarsson, Kasper Malmborn, Anders P. Håkansson

**Affiliations:** Department of Translational Medicine, Division of Experimental Infection Medicine, Lund University, Lund, Sweden

**Keywords:** corynebacteria, microbiota, respiratory tract, pneumonia, *Streptococcus pneumoniae*, inflammation

## Abstract

**Background:**

Certain species from the normal respiratory tract microbiota have recently been proposed to positively influence human health. *Corynebacterium propinquum* and *C. pseudodiphtheriticum* (Corynebacteria) are two Gram-positive species that frequently colonize the upper respiratory tract and strongly associate with a reduced incidence of respiratory tract infections. The specific role of Corynebacteria during respiratory health and disease is, however, largely uncharacterized.

**Method:**

Respiratory tract epithelial cells NCI-H292 and BALB/cByJ mice were inoculated with Corynebacteria (*C. propinquum* 2018M3 and 2019M4, and *C. pseudodiphtheriticum* 2019M8 and 2020M12) alone or with subsequent challenge with *Streptococcus pneumoniae* (pneumococci). The inflammatory response and the bacterial burden of both species over time were determined by Western blot, luciferase assay, cytokine bead array, flow cytometry and viable plate counts on blood agar plates.

**Results:**

Clinical isolates of Corynebacteria were well tolerated by human cells and mice. Corynebacteria induced a transient inflammatory response during healthy conditions in the absence of known pathogens. Pre-exposure or nasal priming with Corynebacteria did not affect subsequent acquisition of pneumococci but were associated with a modulated inflammatory response *in vitro* and *in vivo* as well as with a reduced pneumococcal burden in the respiratory tract of mice. This indicates that the presence of *C. propinquum* or *C. pseudodiphtheriticum* may protect against severe pneumococcal infections.

**Conclusions:**

In this study, we delineate the role of Corynebacteria from the normal microbiota that epidemiologically associate with respiratory health. We show that the presence of Corynebacteria modulates the inflammatory response to pneumococci and associate with faster decrease in pneumococcal burden, primarily in the lower respiratory tract. Our data indicate that Corynebacteria has potential to protect against severe pneumococcal infections.

## Introduction

Lower respiratory tract infection (RTI) is one of the most common causes of morbidity and mortality worldwide with 489 million cases and 2.5 million deaths reported in 2019 (Diseases and Injuries, 2020). *Streptococcus pneumoniae* (the pneumococcus) accounts for more deaths than all other etiologies combined (Collaborators, 2018; Safiri et al., 2022). To cause infection, most respiratory pathogens, including pneumococci, first colonize mucosal surfaces asymptomatically without causing any persistent inflammatory reactions (Hakansson et al., 2018; Drigot and Clark, 2024). In fact, colonization of the nasopharynx (NPH) has been proposed to be a prerequisite for subsequent dissemination to distant sites, resulting in infections such as otitis media, pneumonia, and sepsis (Hakansson et al., 2018; De Steenhuijsen Piters et al., 2022). This “virulence shift” is still incompletely understood, but involves environmental perturbances of the host immune system as well as interactions with other microorganisms in the microbiota (Hakansson et al., 2018).

The mucosal surface of the upper respiratory tract (RT) is colonized by numerous bacterial species, with distinct microbial profiles apparent already at 1.5 month after birth (Biesbroek et al., 2014). The composition of these bacterial communities (the microbiota) varies over time from infancy, where the predominating phyla are Firmicutes, Proteobacteria, Actinobacteria and Bacteroidetes, to puberty, where Actinobacteria and Firmicutes dominate and persist through adulthood (Costello et al., 2009; Bogaert et al., 2011; Biesbroek et al., 2014; Brugger et al., 2016). Recently, it was proposed that the normal respiratory microbiota in the NPH act as a “gate-keeper” of respiratory health and disease (Man et al., 2017). Multiple laboratories have shown that a fluctuating microbial profile with an abundance of *Veillonella*, *Rothia*, *Staphylococcus* or *Haemophilus* species associates with an enhanced risk of developing RTIs (Laufer et al., 2011; Biesbroek et al., 2014; Teo et al., 2015; De Steenhuijsen Piters et al., 2016; Chonmaitree et al., 2017; Lappan et al., 2018). In contrast, a more stable upper RT microbiota, with high abundance of *Corynebacterium* spp. (Corynebacteria) alone or in combination with *Dolosigranulum* spp., is associated with a reduced incidence of RTIs in infants (Pettigrew et al., 2012; Biesbroek et al., 2014; Heberle et al., 2015; Chonmaitree et al., 2017; Lappan et al., 2018). Corynebacteria are similarly abundant in the NPH of healthy immuno-competent adults, and the presence of specific Corynebacteria species associates inversely with the presence of pneumococci (Laufer et al., 2011; Pettigrew et al., 2012; Xu et al., 2021; Paulo et al., 2023).

A mechanistic understanding of the positive influence of Corynebacteria on human health is still limited but both direct competition with potential pathogens and modulation of the immune system have been proposed (De Steenhuijsen Piters et al., 2015). *C. accolens*, *C. propinquum* and *C. pseudodiphtheriticum* can inhibit pneumococcal growth *in vitro*, partly via release of inhibitory fatty acids (Bomar et al., 2016; Xu et al., 2021). Furthermore, nasal priming with *C. pseudodiphtheriticum* strain 090104 was recently proposed to protect mice against respiratory syncytial virus (RSV)-infection and secondary pneumococcal pneumonia through unknown mechanisms possibly related to strain-specific immunomodulation (Kanmani et al., 2017; Ortiz Moyano et al., 2020). The protective properties of Corynebacteria are further complicated by the fact that the microbiota exerts niche-specific effects (Zheng et al., 2020). We recently reported that the presence of *C. propinquum* associates with pro-inflammatory mediators in middle ear effusions from children with otitis media, but with anti-inflammatory mediators in the corresponding NPH samples (Enoksson et al., 2020). Thus, the potential immunomodulatory role of Corynebacteria in the RT is complex and largely uncharacterized.

In this study, we aimed to investigate the role of Corynebacteria in the RT over time, focusing on the interaction between Corynebacteria and the inflammatory response, as well as whether Corynebacteria may modulate the inflammatory response to pneumococci.

## Materials and methods

### Bacterial strains

The clinical isolates *Corynebacterium propinquum* 2018M3 (Cp1) and 2019M4 (Cp2), and *C. pseudodiphtheriticum* 2019M8 (Cps1) and 2020M12 (Cps2) from the RT normal microbiota of healthy infants were kindly provided by Dr. Debby Bogaert, University of Edinburgh, UK (Biesbroek et al., 2014). Corynebacteria were grown in brain heart infusion (BHI; Sigma Aldrich) overnight at 37°C, aerated by shaking at 180rpm. *Streptococcus pneumoniae* lab strain D39 [serotype 2 (Avery et al., 1944)] was grown statically in Todd Hewitt broth supplemented with 0.5% yeast extract (THY; Sigma Aldrich) at 37°C until they reached an OD_600_ of 0.3-0.6. For cell stimulation experiments, all strains were diluted to an optical density at 600nm (OD_600_) of 0.1 (approximately 10^6^ CFU/ml for both Corynebacteria and D39), washed in PBS, and resuspended in supplement-free cell culture medium (see below). For *in vivo* challenge experiments, bacteria were concentrated by centrifugation and were saved as frozen stocks at -80°C in 15% glycerol.

### *In vitro* interaction assays between Corynebacteria and D39 pneumococci

1) For indirect interaction assays, Corynebacteria and D39 pneumococci were grown until log-phase and supernatants were collected by two rounds of centrifugation at 3500 rpm, 10 min, followed by sterile filtration (0.22μm) to further remove bacteria. All supernatants were free of viable cells, as determined by plating on blood agar plates. Fresh medium or cell-free supernatants (500 μl) was then allowed to absorb onto blood agar plates before plating serial dilutions of bacteria to determine the bacterial burden (of both Corynebacteria and pneumococci). The plates were incubated over night at 37°C. 2) For direct interaction assays, 5μl spots of Corynebacteria and D39 pneumococci from liquid cultures were inoculated onto blood agar plates alone, directly adjacent or with 1 cm apart. The plates were incubated up to 72h at 37°C, with image analyses performed at 24, 48 and 72h. The diameter and area of the colonies were determined using ImageJ (NIH, MD, USA) and normalized against the diameter of the plate to adjust for differences between images.

### Respiratory tract epithelial cells

Cell culture media, supplements and trypsin were all from Saveen-Werner. The mucoepidermoid bronchial carcinoma cell line NCI-H292 (ATCC CCL-1848) was routinely grown at 37°C, 5% CO_2_ in RPMI-1640 (with L-glutamine) supplemented with 10% heat-inactivated FBS, 100U/ml penicillin, 100μg/ml streptomycin and 1mM sodium pyruvate. Cells were seeded into 24-well plates and grown until confluent. The cells were then incubated in RPMI-1640 without supplements and allowed to adjust to the nasopharyngeal temperature of 34°C overnight (Keck et al., 2000).

### *In vitro* cell stimulations

Live cell monolayers were washed once in PBS and stimulated with 0.5ml of bacterial suspensions. Stimulations with 100ng/ml or 1μg/ml of lipopolysaccharide (LPS; from *Salmonella enterica* serotype typhimurium, Sigma Aldrich; St Louis MO, USA) were used as controls for general inflammatory responses. All stimulations were performed at 34°C, 5% CO_2_, to mimic the nasopharyngeal temperature (Keck et al., 2000), in cell culture medium without supplements, and samples were collected at indicated time points. For stimulations longer than 24h, the medium was replaced every 24h.

### Cell viability assessment

The viability of NCI-H292 cells was assessed by trypan blue exclusion assay (ThermoFisher Scientific) or by Annexin V and propidium iodide (PI) staining (BD Biosciences), according to the manufacturers’ instructions. For Annexin V/PI analyses, the percentage of viable cells was determined as AnnexinV^-^ PI^-^ cells using a FACSCalibur (BD Biosciences) and analyzed by the FlowJo software (version 8.8.7).

### Dual luciferase reporter assay

NCI-H292 cells were transfected with 0.05μg pTK-Renilla luciferase vector (Promega) as a control, together with 0.5μg pNFκB-luciferase plasmid (BD Biosciences) using the Turbofect transfection reagent (ThermoFisher Scientific) according to the manufacturer’s instructions. The cells were stimulated 24h post-transfection and harvested at indicated time points. Relative luciferase activity (RLU) was determined using Dual-Luciferase assay (Promega) using the Synergy 2 microplate reader (BioTek, Winooski, VT).

### Western blot

NCI-H292 cells stimulated with bacteria, LPS or medium alone were washed in PBS and immediately lysed in boiling Laemmli sample buffer supplemented with 100 mM DTT. Lysates were separated on 10% SDS-PAGE gels, transferred to PVDF membranes, and blotted using the following primary antibodies: ERK1/2-P (Thr202/Tyr204, clone 197G2) and ERK1/2 (clone 137F5) from Cell signaling (Danvers, MA, USA), IκBα (clone H-4) from Santa Cruz and Actin (clone C-4) from MP Biomedicals (Solon, OH, USA). After addition of HRP-conjugated secondary antibody (from MP Biomedicals) and chemiluminescent substrate (Amersham), the blots were analyzed using a CCD camera (BioRad, Chemidoc MP). Densitometry of the bands was performed using ImageJ (NIH, MD, USA).

### *In vivo* animal procedures

Ethical permit for *in vivo* experiments was obtained from the Regional Ethics Committee in Lund (Dnr 08146/2017). i) For aspiration of bacteria into the NPH and lungs, 6–8-week-old female BALB/cByJ mice (Janvier Labs) were lightly anesthetized using isofluorane and inoculated intranasally with 10^8^ CFU/mouse of Corynebacteria and/or 2x10^7^ CFU/mouse of D39 pneumococci in 40μl volume. Sterile PBS (40μl) was used as control. ii) For intranasal priming, 20μl of Corynebacteria (10^8^ CFU/mouse) or 20μl of sterile PBS was intranasally administered to non-anesthetized mice on five consecutive days. The dose of 10^8^ CFU/mouse of Corynebacteria as well as the selection of five consecutive days for intranasal priming, were chosen based on a previous study using 10^8^ CFU/mouse/day of *C. pseudodiphtheriticum* 090104 (Kanmani et al., 2017). Corynebacteria- or PBS-inoculated mice were then left untreated or were lightly anesthetized and subjected to aspiration of either 40μl of D39 suspension (2x10^7^ CFU/mouse) or 40μl of sterile PBS.

Mice were monitored daily for signs of illness (e.g., ruffled fur or lethargy). At indicated time points (4h, 24h, 48h, 72h post inoculation), the mice were sacrificed by CO_2_-inhalation. Bronchoalveolar lavage (BAL) was performed with 200μl of sterile PBS and nasal lavage (NAL) with 100μl of sterile PBS, through a small incision in the trachea. Tissue was harvested as described (Chao et al., 2019). Briefly, the lung lobes were excised by opening the thoracic cavity. To collect NPH tissue, the nose and skull of the mouse was defleshed using scissors. The maxillary bones and the skull between the eyes were cut and the tissue in the nasal conchae was collected with forceps.

### *In vivo* sample processing

Lungs and NPH tissues were mechanically homogenized in 1ml of sterile PBS. The bacterial burden (Corynebacteria and pneumococci) in tissue homogenates as well as BAL and NAL samples was determined by plating serial dilutions on blood agar plates and counting colonies after overnight incubation. Corynebacteria and pneumococcal colonies were distinguished based on colony morphology (Corynebacteria; raised, white, opaque colonies and pneumococci; flat, green colonies with hemolysis). The remaining BAL and NAL samples were pelleted at 1,200rpm for 5min and the supernatants were collected and stored at -80°C for analyses of inflammatory mediators (see below). After red blood cell lysis with ACK lysis buffer (ThermoFisher), the number of viable cells in pellets was determined by trypan blue exclusion and samples with >4,000 viable cells were analyzed by flow cytometry (see below). Homogenized lung and NPH tissues were further dissociated by collagenase type I treatment (Fisher Scientific; 210μl of 200mg/ml stock solution added to 15-20ml of cell culture medium) for 1h at 37°C. Tissue samples were then subjected to red blood cells lysis, filtered through a 100μm cell strainer to obtain a single cell suspension and analyzed by flow cytometry (see below).

### Flow cytometry

Cells from NAL, BAL, lung and NPH tissues were washed in FACS buffer (PBS with 3% FBS and 0.1% sodium azide) and stained using the following antibodies (all from BD Biosciences), with clones and dilutions indicated: CD45-FITC (clone 30-F11, 1:30), CD3e-APC (clone 17A2, 1:30), CD19-PE-Cy7 (clone 1D3, 1:25), CD335/NKp46-PE (clone 29A1.4, 1:25), Ly6G-APC (clone 1A8, 1:40), CD11c-PE-Cy7 (clone HL3, 1:25) and CD64-PE (clone X54-5/7.1, 1:30), and using 7AAD as a cell death discriminator. The cells were analyzed using a FACSVerse (BD Biosciences) and the FlowJo software.

### Cytokine analyses

Supernatants from NCI-H292 cells were analyzed using the Human Inflammatory Cytokine Bead Array kit (CBA; BD Biosciences). NAL and BAL samples from mice were analyzed using the Mouse Inflammation CBA kit (BD Biosciences). The concentrations of indicated mediators were determined using FACSVerse and the FlowJo software. The concentrations of human IL-1β, IL-10, TNFα and IL-12 were generally below the detection limit and were excluded from further analyses.

### Statistical analyses

Statistical analyses were made using GraphPad Prism 10 Statistics Software (La Jolla, CA, USA). One-way ANOVA with Dunnett’s multiple comparison test was used for comparison of more than two groups to one control. One-way ANOVA with Tukey’s or Sidak’s multiple comparison tests were used as indicated for comparisons between more than two samples (e.g., for comparison between time points within the same treatment group and niche, or between treatment groups at the same time point and niche). Unpaired two-tailed t-test was used to compare two groups. *P*-values <0.05 were considered significant.

## Results

### Corynebacteria induce inflammatory signaling *in vitro*

We first investigated the effect of Corynebacteria alone on RT epithelial cells. Two clinical isolates each of *C. propinquum* (Cp1 and Cp2) and *C. pseudodiphtheriticum* (Cps1 and Cps2), that epidemiologically associate with reduced incidence of RTIs (Pettigrew et al., 2012; Biesbroek et al., 2014), were inoculated onto the RT epithelial cell line NCI-H292. In line with their role as normal microbiota, we observed no major effect on NCI-H292 cell viability after 24h, but reduced cell viability was observed at 72h (**Supplementary Figures 1A–C**).

**Figure 1:**
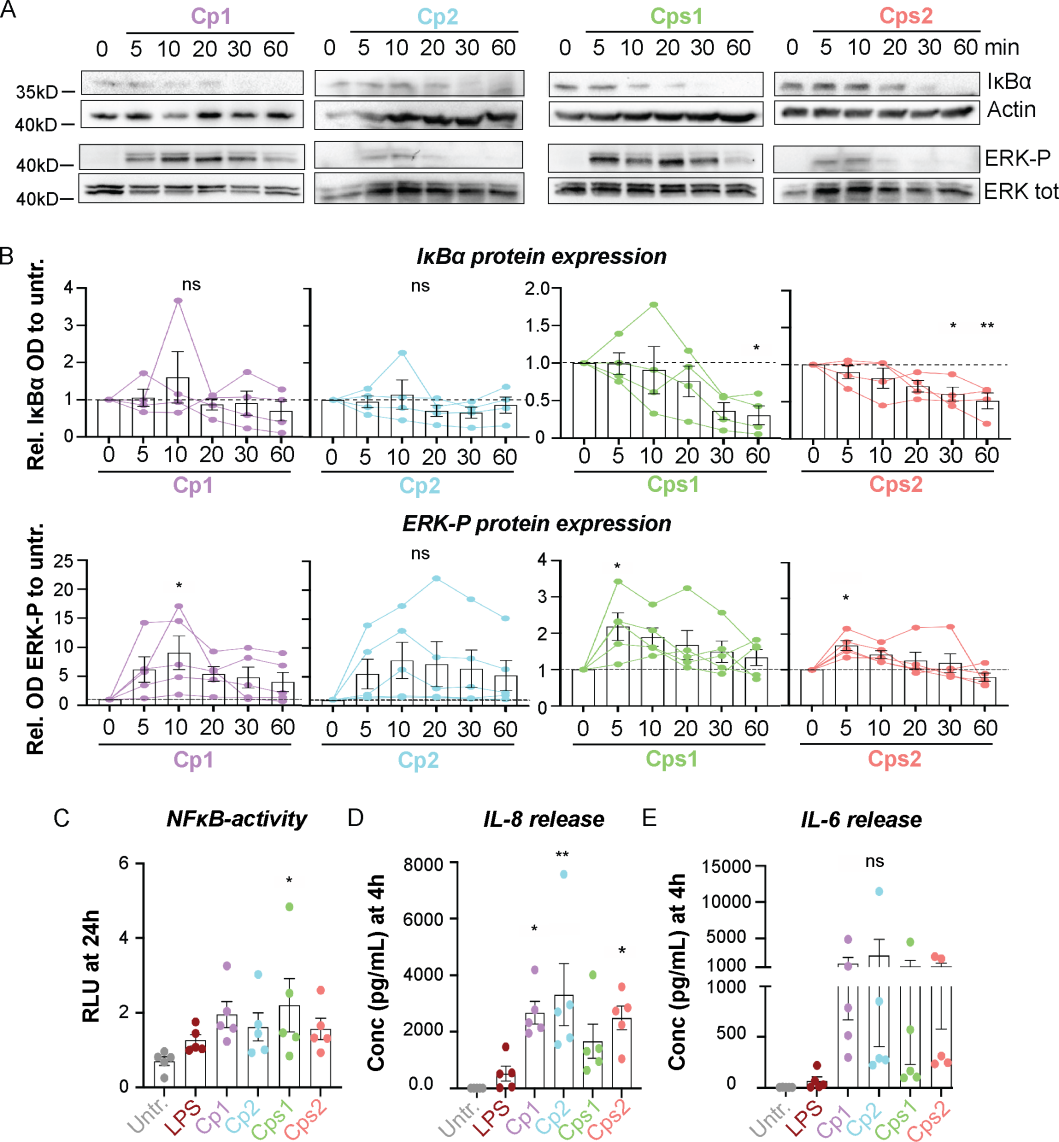
Corynebacteria induce inflammatory signaling in NCI-H292 cells. Confluent NCI-H292 cells were stimulated with *C. propinquum* (Cp1 and Cp2) or *C. pseudodiphtheriticum* (Cps1 and Cps2) or LPS in serum-free medium (SFM) for indicated time points. **(A, B)** NFκB- and ERK-signaling was assessed by Western blot by determining the degradation of inhibitor of kappa B alpha (IκBα) and phosphorylation of ERK (ERK-P), respectively. Actin and total ERK were used as controls. Representative blots **(A)** and mean relative OD IκBα (**B**, *upper panel*) or OD ERK-P (**B**, *lower panel*) to untreated control (0min; dashed lines), with individual data points, mean and SEM shown from N=4-5 experiments. **(C)**. The NFκB activity in NCI-H292 cells stimulated with LPS or Corynebacteria for 24h. The relative dual luciferase units (RLU) were measured using TK-Renilla as control. Graphs represent individual values with mean and SEM from N=5 individual experiments. **(D–E)** The concentration of IL-8 **(D)** and IL-6 **(E)** in supernatants from NCI-H292 cells stimulated with Corynebacteria or LPS for 4h was determined by cytokine bead array (CBA). Values below the detection limit of 3.6 pg/mL and 2.5 pg/mL for IL-8 and IL-6, respectively, were put to the detection limit. Individual values with mean and SEM shown from N=5 experiments. All statistics by ordinary one-way ANOVA with Dunnett’s multiple comparison test to the untreated control, ns, not significant, * *P* < 0.05, ** *P* < 0.01.

We next analyzed the host response to Corynebacteria and compared it to the stimulating effects of LPS. All Corynebacteria strains induced moderate activation of NFκB and ERK-signaling in NCI-H292 cells, as determined by Western blot (**Figures 1A, B**). NFκB activity was also analyzed by luciferase assay, where Corynebacteria-induced NFκB-activity remained high up to 72h and was slightly higher than or comparable to the activity induced by LPS (**Figure 1C**; **Supplementary Figure 1D**). In line with the observed NFκB-activation, Corynebacteria induced release of inflammatory mediators IL-8 and IL-6 (**Figures 1D, E**; **Supplementary Figures 1E,F**). Corynebacteria induced significantly higher levels of IL-8 from 4h to 48h and of IL-6 at 24h after stimulation compared to untreated and LPS stimulated cells (**Figures 1D, E**; **Supplementary Figures 1E, F**). These results indicate that Corynebacteria induce an inflammatory response in RT epithelial cells.

### Transient colonization of Corynebacteria in the mouse NPH

**Figure 2:**
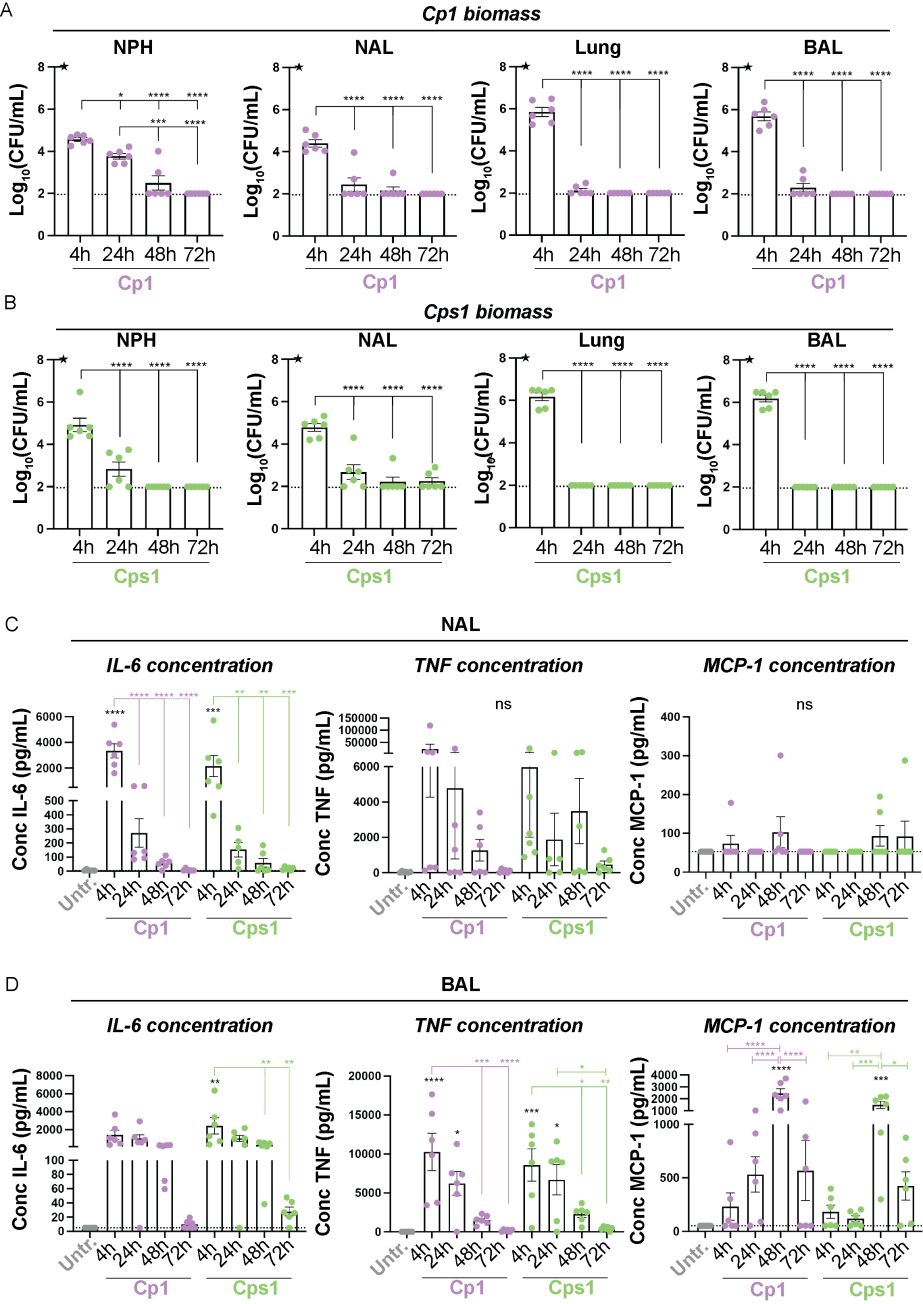
Corynebacteria persist up to 48h in the nasopharynx and induce strain and niche-specific release of inflammatory mediators. Corynebacteria were intra-nasally administered to lightly anesthetized BALB/cByJ mice at 10^8^ CFU/mouse (initial inoculum; indicated by black stars) to allow aspiration of Corynebacteria into the respiratory tract. The mice were sacrificed at indicated time points and nasopharyngeal tissue (NPH), nasal lavage (NAL), lung tissue (lung) and bronchoalveolar lavage (BAL) were collected and analyzed. Untreated mice (untr) served as controls. **(A, B)** The biomass of Corynebacteria *C. propinquum* (Cp1; **A**) *C. pseudodiphtheriticum* (Cps1; **B**) was determined at the distinct niches for each time point by viable plate counts on blood agar plates. Not detectable values were put to the detection limit (1.996 log_10_ CFU/mL; dotted line). Statistics by ordinary one-way ANOVA with Tukey’s multiple comparison test between the time points within each niche. **(C, D)** The concentration (pg/mL) of IL-6 (*left panels*), TNF (*middle panels*) and MCP-1 (*right panels*) were determined in cell-free NAL **(C)** and BAL **(D)** samples by cytokine bead array (CBA). Values below the detection limit were put to the detection limit (dotted lines; 5 pg/mL, 7.3 pg/mL and 52.7 pg/mL for IL-6, TNF, and MCP-1, respectively). Graphs represent individual values (mice) with mean and SEM from n=5-6 mice, from N=2 experiments. Statistics by ordinary one-way ANOVA with Sidak’s multiple comparison test to untreated (black asterisks over bars) or over time within treatment (colored asterisks over colored lines). ns, not significant, * *P* < 0.05, ** *P* < 0.01, *** *P* < 0.001 and **** *P* < 0.0001.

To investigate the ability of Corynebacteria to survive in and colonize the RT *in vivo*, BALB/cByJ mice were lightly anesthetized and intranasally inoculated with *C. propinquum* Cp1 or *C. pseudodiphtheriticum* Cps1 (10^8^ CFU/mouse). This procedure enabled aspiration of bacteria into both the nares and lungs. All mice appeared unaffected by the treatment, as indicated by the absence of ruffled fur, isolation, and general behavior. At indicated time points after inoculation, NPH tissue, nasal lavage (NAL), lung tissue and bronchoalveolar lavage (BAL) were harvested. Corynebacteria persisted in the NPH up to 48h post-inoculation but were only detectable up to 4h (Cps1) and 24h (Cp1) in the lungs (**Figures 2A, B**). This indicates that Corynebacteria transiently colonize the mouse NPH.

### Corynebacteria induce strain and niche-specific release of inflammatory mediators *in vivo*

Next, we analyzed the levels of inflammatory mediators in NAL and BAL samples from mice inoculated with Corynebacteria and compared them with samples from untreated mice or from PBS alone (vehicle)-treated mice. Overall, NAL and BAL samples from untreated and vehicle-inoculated mice were similar, with the levels of all mediators being close to or below the limit of detection (**Figures 2C, D**, **Supplementary Figure 2**). One exception was that aspiration of vehicle-alone induced a modest release of IL-6 and TNF in NAL and BAL samples at 4h that was above those detected in untreated mice, although still 10-100-fold lower than in mice inoculated with Corynebacteria (**Supplementary Figures 2A, B**; **Figures 2C, D**). Thus, vehicle-inoculation induced a minor release of specific inflammatory mediators in NAL and BAL samples compared to untreated controls.

NAL from mice inoculated with Corynebacteria showed levels of MCP-1, IFNγ, IL-12 and IL-10 close to or below the limit of detection (**Figure 2C**, and data not shown). On the other hand, IL-6 and TNF-levels were highly elevated at 4h after inoculation with Corynebacteria compared to untreated mice but then declined over time and returned to levels close to those of untreated mice (**Figure 2C**).

In BAL samples, IL-6, TNF, MCP-1, IFNγ, IL-12 and IL-10 levels were generally higher in Corynebacteria-inoculated mice than in untreated mice (**Figure 2D**; **Supplementary Figures 3A–C**). Similar to NAL samples, the concentrations of IL-6 and TNF in BAL peaked at 4h after inoculation with Corynebacteria, whereas MCP-1 levels peaked at 48h after inoculation and were significantly higher than in BAL from untreated mice (**Figure 2D**). Although the kinetics and the pattern of inflammatory mediators induced by *C. propinquum* (Cp1) and *C. pseudodiphtheriticum* (Cps1) were close to identical, the concentration of IFNγ and IL-12 tended to be higher in BAL from Cps1- compared to Cp1-inoculated mice, although only significantly so for IL-12 at 24h (**Supplementary Figures 3A–C**). In contrast, IL-10 was induced 4-24h after inoculation with Cp1 but not Cps1 and was significantly higher than in BAL samples from untreated mice or Cps1 treated mice at 24h (**Supplementary Figure 3C**). Combined, these results indicate that Corynebacteria induce similar, yet strain-specific, release of inflammatory mediators that differs between the niches (NAL *versus* BAL samples).

### Aspiration of Corynebacteria affect the immune landscape in the respiratory tract

To determine the immune cell composition at the distinct niches (NPH, NAL, lung and BAL), all samples with >4000 viable cells were analyzed by flow cytometry and reported as percentages of all viable CD45^+^ leukocytes. Consequently, all findings regarding immune cell populations below should be considered collectively as an increase in the percentage of one population inherently results in a decrease in the percentage of another. Representative gating strategies are presented in **Supplementary Figure 3D**. PBS-inoculated (vehicle) mice displayed a similar percentage of CD45^+^ leukocytes and immune cells as untreated mice at all niches (**Supplementary Figure 2C** and data not shown).

**Figure 3:**
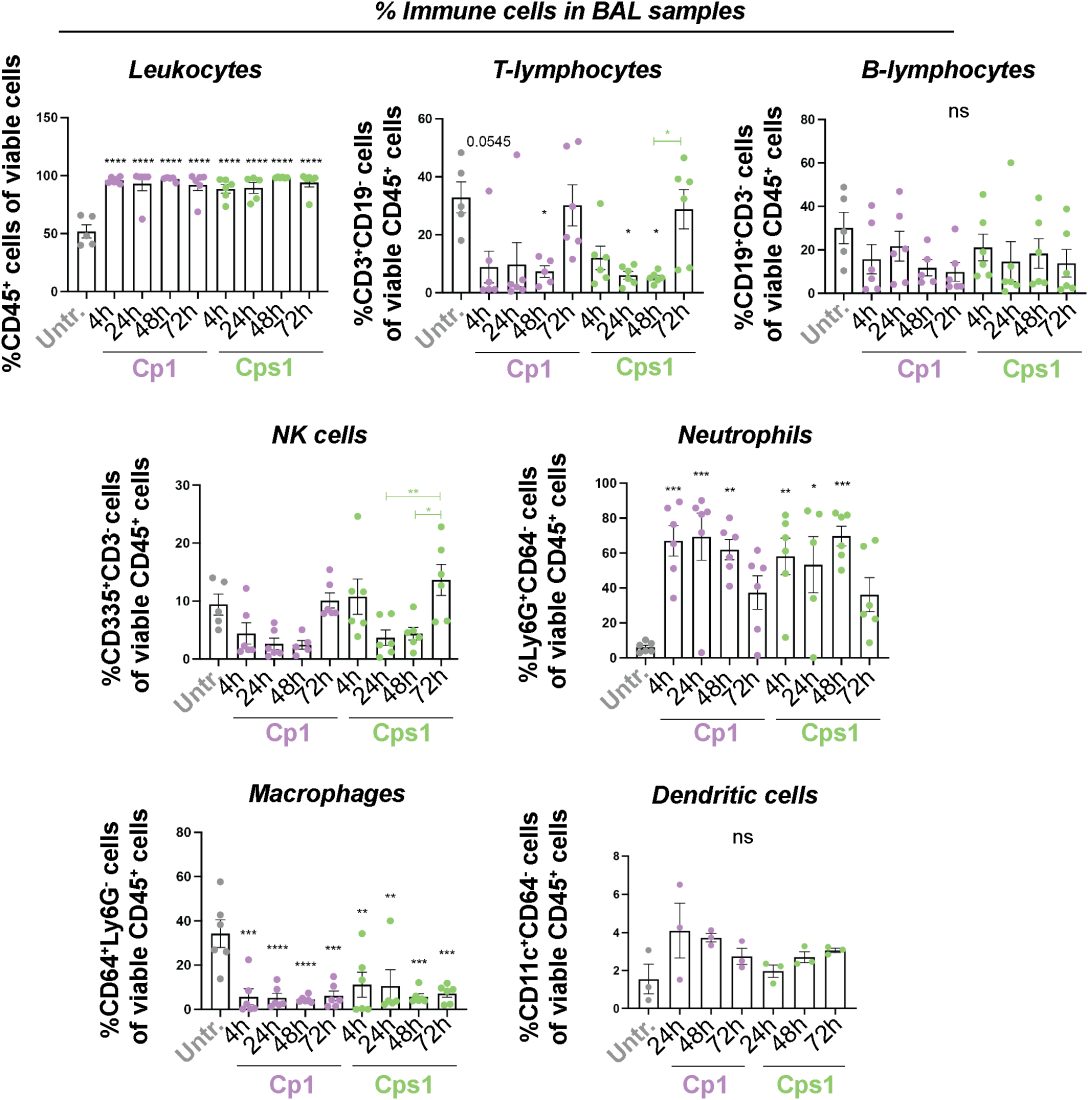
Aspiration of Corynebacteria induce changes in the immune cell composition in BAL samples. Corynebacteria were intra-nasally administered to lightly anesthetized BALB/cByJ mice at 10^8^ CFU/mouse to allow aspiration of Corynebacteria into the respiratory tract. Untreated mice (untr) served as controls. The mice were sacrificed at indicated time points and bronchoalveolar lavage (BAL) were collected and analyzed. The percentage of the respective immune cell population was determined by flow cytometry in pelleted lavage samples with > 4000 viable cells per sample as determined by trypan blue exclusion assay. Leukocytes; CD45^+^ cells of all viable (7AAD^-^) cells, T-lymphocytes; CD3^+^CD19^-^ cells of all viable CD45^+^ cells, B-lymphocytes; CD19^+^CD3^-^ cells of all viable CD45^+^ cells, NK cells; CD335^+^ CD3^-^CD19^-^ cells of all viable CD45^+^ cells, neutrophils; Ly6G^+^CD64^-^ cells of all viable CD45^+^ cells, macrophages; CD64^+^Ly6G^-^ cells of all viable CD45^+^ cells and dendritic cells; CD11c^+^CD64^-^ cells of all viable CD45^+^ cells. Graphs represent individual values (mice) with mean and SEM from n=3-6 mice from N=2 experiments. Statistics by ordinary one-way ANOVA with Sidak’s multiple comparison test to untreated (black asterisks over bars) or over time within treatment (colored asterisks over colored lines). ns, not significant, * *P* < 0.05, ** *P* < 0.01, *** *P* < 0.001 and **** *P* < 0.0001.

Apart from a significant reduction in the percentage of CD64^+^Ly6G^-^ macrophages in NPH samples (**Supplementary Figure 4A**), aspiration of Corynebacteria induced only minor alterations in the percentages of immune cell populations analyzed from NAL and NPH samples (**Supplementary Figure 4**). In BAL samples, however, mice inoculated with Corynebacteria displayed significant enrichment of CD45^+^ leukocytes and Ly6G^+^CD64^-^ neutrophils compared to untreated mice already at 4h, that persisted over 72h (**Figure 3**). Conversely, the levels of CD3^+^CD19^-^ T-lymphocytes and CD64^+^Ly6G^-^ macrophages were significantly lower in BAL samples from mice inoculated with Corynebacteria compared to untreated mice at 4-48h (**Figure 3**), but returned to the levels of untreated mice at 72h when the neutrophil levels decline. Similar trends were observed for CD19^+^CD3^-^ B-lymphocytes and CD335^+^CD3^-^CD19^-^ NK cells (**Figure 3**).

The changes in immune cell composition were less pronounced in lung tissue samples. Mice inoculated with Corynebacteria displayed higher levels of CD45^+^ leukocytes, Ly6G^+^CD64^-^ neutrophils and CD64^+^Ly6G^-^ macrophages compared to untreated mice, although only significantly so for neutrophils (**Supplementary Figure 5**). The levels of CD3^+^CD19^-^ T-lymphocytes, and CD335^+^CD3^-^ NK cells were similar, whereas the levels of CD19^+^CD3^-^ B-lymphocytes and CD11c^+^CD64^-^ dendritic cells tended to be lower in the lungs of Corynebacteria-inoculated mice compared to untreated mice (**Supplementary Figure 5**).

Combined, these results indicate that aspiration of Corynebacteria only induce minor changes in the immune landscape in the upper RT. In contrast, more distinct changes were observed in the lower RT (predominantly in BAL samples) after inoculation with Corynebacteria, with higher levels of most inflammatory mediators investigated as well as higher levels of neutrophils and accompanying lower levels of T-lymphocytes. It should be noted that the lung tissues were not perfused to remove blood prior to analyses and therefore contain immune cells from the blood as well as from the tissue. This, combined with the preferential presence of specific tissue resident and recruited immune cells in BAL may explain the differences in BAL and lung tissue samples.

### No *in vitro* effect of Corynebacteria on pneumococcal viability or growth or vice versa

We next sought to investigate the role of Corynebacteria during infection with *S. pneumoniae* (pneumococci) *in vitro*. Supernatants from *C. accolens* and *C. amycolatum* have previously been shown to inhibit pneumococcal growth *in vitro* (Horn et al., 2021). Similarly, *C. propinquum* and *C. pseudodiphtheriticum* inhibited the growth of a pneumococcal serotype 22F strain when spotted on blood agar plates (Xu et al., 2021). To investigate the potential ability of Corynebacteria clinical isolates to inhibit pneumococcal viability or growth, we pre-treated blood agar plates with either medium (control) or cell-free supernatants from Corynebacteria cultures, followed by serial dilutions of D39 pneumococci (**Supplementary Figure 6A**). We also pre-treated blood agar plates with cell-free supernatants from D39 cultures and performed serial dilutions of Corynebacteria (**Supplementary Figure 6B**) to investigate the effects of pneumococcal products on Corynebacteria viability and growth. However, Corynebacteria supernatants exerted no inhibitory effect on the growth of pneumococci, or vice versa (**Supplementary Figures 6A, B**). Similarly, when Corynebacteria and D39 pneumococci were inoculated directly adjacent or with 1 cm distance on blood agar plate, no significant differences were observed with regards to colony area compared to bacteria inoculated alone (**Supplementary Figures 6C–E**).

This indicates that neither supernatants from the clinical isolates of Corynebacteria used in this study, nor the proximity of live Corynebacteria, had any effect on the viability or growth of D39 pneumococci under these conditions *in vitro*.

### The presence of Corynebacteria modulates the release of inflammatory mediators in response to pneumococci *in vitro*

In contrast to the high viability observed when stimulating NCI-H292 cells with Corynebacteria alone (**Supplementary Figures 1A–C**), the cell viability was considerably lower after stimulation with D39 pneumococci (**Supplementary Figures 7A–C**). The presence of Corynebacteria tended to protect against the toxic effect of D39 at earlier time points (>50% viability compared to <10% after stimulation with D39 alone at 48h post Corynebacteria-inoculation with D39 added the last 24h) (**Supplementary Figure 7C**). This indicates that although Corynebacteria potentially protect against the cytotoxic effect of pneumococci *in vitro*, shorter stimulations with D39 are required to maintain cell viability.

**Figure 4:**
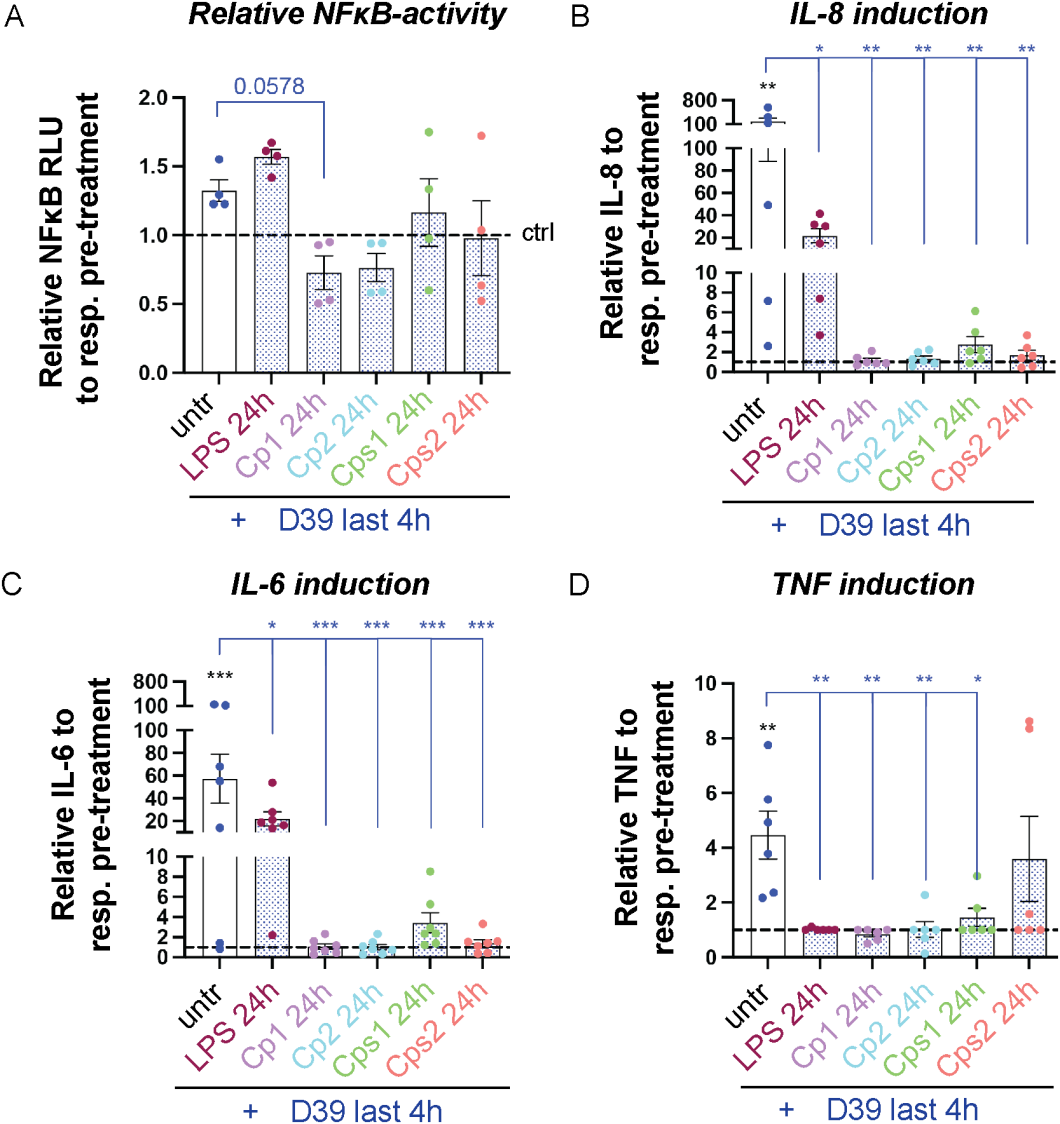
Corynebacteria modulate the inflammatory response to pneumococci *in vitro*. Confluent NCI-H292 cells were either left unstimulated or pre-stimulated with 100 ng/mL LPS, Corynebacteria *C. propinquum* (C1 and C2) or *C. pseudodiphtheriticum* (Cps1 and Cps2) in serum-free medium (SFM) for 24h. D39 pneumococci were added the last 4h. The values from the respective pre-treatment control (ctrl; only medium, LPS or Corynebacteria) were put to 1 (dashed lines). **(A)** The relative NFκB activity to the respective pre-treatment control was determined by dual luciferase assay using TK-Renilla as control. **(B–D)** The relative induction of IL-8 **(B)**, IL-6 **(C)** and TNF **(D)** in supernatants from D39-treated cells pre-treated or not with LPS or Corynebacteria was determined by cytokine bead array (CBA). Values below the detection limit of 3.6 pg/mL, 2.5 pg/mL and 3.7 pg/mL for IL-8, IL-6 and TNF, respectively, were put to the detection limit. Individual values with mean and SEM shown from N=4-7 experiments. Statistics by ordinary one-way ANOVA with Dunnett’s multiple comparison test to untreated (black asterisks) or D39 only (without pre-treatment; blue asterisks over blue bars). * *P* < 0.05, ** *P* 0.01, *** *P* < 0.001.

To study whether Corynebacteria modulate the inflammatory response triggered by pneumococci *in vitro*, NCI-H292 cells were pre-stimulated with Corynebacteria for 24h and subsequently stimulated with D39 pneumococci for a short duration (4h). Pre-stimulation with LPS (to model a general ongoing inflammatory response) or medium alone (untreated, *i.e.*, representing no pre-treatment) served as controls. Stimulation with D39 alone resulted in a modest, non-significant, increase in NFκB -activity (**Figure 4A**; **Supplementary Figure 7D**). Pre-treatment with LPS slightly enhanced the D39-induced NFκB-activity (**Figure 4A**; **Supplementary Figure 7D**), whereas cells pre-treated with Corynebacteria showed NFκB-activity that were similar whether or not the cells were subsequently inoculated with D39 (**Supplementary Figure 7D**). Consequently, the relative NFκB-activity after D39 treatment was unaffected or lower in the presence of Corynebacteria compared to cells pre-treated with LPS or in cells only treated with D39, although not significantly so (**Figure 4A**).

In parallel, we investigated the release of inflammatory mediators in supernatants from untreated NCI-H292 cells and cells pre-treated with LPS or Corynebacteria for 24h alone or after additional treatment with D39 for 4h (**Figures 4B–D**; **Supplementary Figures 7E–G**). All treatments induced non-significant absolute release of IL-8, IL-6 and TNF compared to untreated cells (**Supplementary Figures 7E–G**). Corynebacteria- or LPS-pretreated cells displayed significantly lower relative release of IL-8, IL-6 and TNF after D39 treatment compared to cells treated with D39 alone (no pre-treatment; **Figures 4B–D**). In fact, no significant additional induction of cytokine release was seen after D39 stimulation of cells that had been pretreated with Corynebacteria (**Figures 4B–D**). This can be attributed to the overall high level of release of inflammatory mediators observed from cells pre-treated with Corynebacteria regardless of D39 treatment (**Supplementary Figures 7E–G**).

Altogether, these results indicate that Corynebacteria induce inflammatory responses that predominate to the extent that subsequent treatment with pneumococci results in no major increase in inflammation or results in a diminished induction of inflammatory responses.

### Pre-exposure of mice to Corynebacteria decrease the pneumococcal burden in the lungs

**Figure 5:**
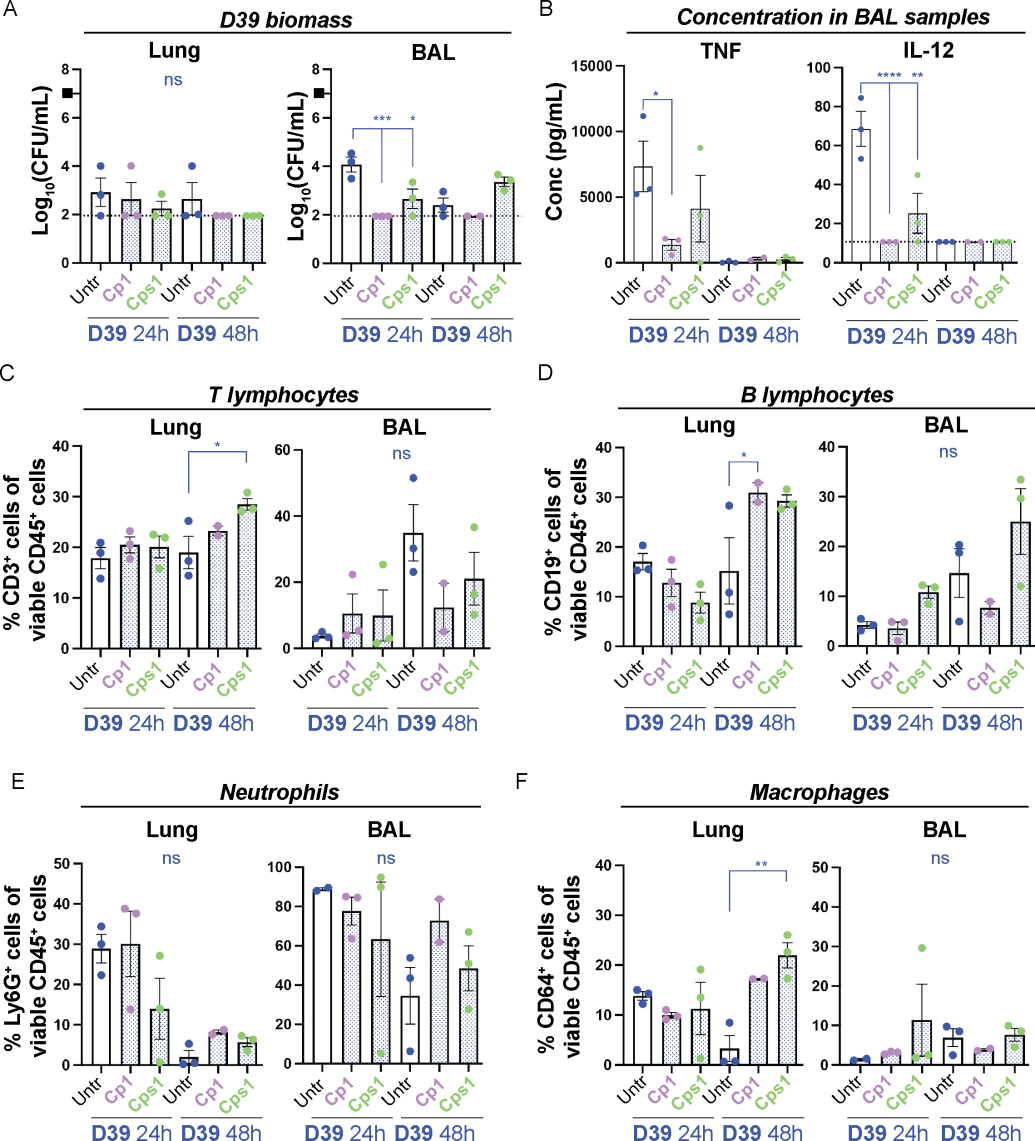
Pre-exposure of mice with Corynebacteria associate with decreased pneumococcal burden and a modulated immune response. Corynebacteria (*C. propinquum* Cp1 and *C. pseudodiphtheriticum* Cps1) were intranasally administered to lightly anesthetized BALB/cByJ mice at 10^8^ CFU/mouse to allow aspiration of Corynebacteria. After 24h, the mice aspirated 10^7^ CFU/ml D39 pneumococci (indicated by the black box). Lung tissue (lung) or bronchoalveolar lavage (BAL) samples were collected 24 or 48h after inoculation with D39. Additional information regarding nasopharynx tissue (NPH), nasal lavage (NAL), lung and BAL samples is presented in **Supplementary Figure 8**. **(A)** The bacterial burden of D39 pneumococci was determined by viable plate count on blood agar plates. The biomass (log_10_ CFU/mL) of D39 from mice pre-exposed to Cp1 or Cps1 with individual values, mean and SEM shown. Values below the detection limit of 1.95 log_10_ CFU/mL were put to the detection limit (dotted line). The biomass of Corynebacteria was generally close to or below the detection limit (2-3 log_10_ CFU/mL) in all samples investigated and not shown. **(B)** The concentration of TNF (*left panel*) and IL-12 (*right panel*) was determined by cytokine bead array (CBA) in cell-free BAL samples. Values below the detection limit of 7.3 pg/mL and 10.7 pg/mL for TNF and IL-12, respectively, were put to the detection limit (dotted lines). **(C–F)** The percentage of the respective immune cell population was determined by flow cytometry in homogenized lung tissue or pelleted BAL samples with > 4000 viable cells per sample as determined by trypan blue exclusion assay: CD3^+^CD19^-^ T-lymphocytes of all viable CD45^+^ cells **(C)**, CD19^+^CD3^-^ B lymphocytes of all viable CD45^+^ cells **(D)**, Ly6G^+^CD64^-^ neutrophils of all viable CD45^+^ cells **(E)**, CD64^+^Ly6G^-^ macrophages of all viable CD45^+^ cells **(F)**. Individual values (mice) with mean and SEM shown from n=3 mice, N=1 experiment. All statistics by ordinary one-way ANOVA with Sidak’s multiple comparison test to D39 only without pre-exposure to Corynebacteria (blue asterisks over blue lines). ns, not significant, * *P*< 0.05, ** *P* < 0.01, *** *P* < 0.001, **** *P* < 0.0001.

To investigate whether the presence of Corynebacteria in the RT affects the acquisition or persistence of pneumococci or modulate the host inflammatory response *in vivo*, mice were pre-inoculated with Corynebacteria (*C. propinquum* Cp1, and *C. pseudodiphtheriticum* Cps1) by aspiration into nares and lungs, and were then inoculated with D39 pneumococci 24h later. The bacterial burden in NPH, NAL, lung and BAL samples was determined 24h or 48h after inoculation with D39. The biomass of Corynebacteria was generally below the limit of detection in all samples and at all time points. The presence of Corynebacteria had little to no effect on the D39 biomass in the NPH, NAL and lung tissue samples (**Figure 5A**; **Supplementary Figure 8A**). However, in BAL samples, lower levels of D39 were observed from mice pre-inoculated with Cp1 or Cps1 compared to mice only inoculated with D39 (**Figure 5A**).

This indicates that pre-exposure to Corynebacteria does not affect the acquisition of pneumococci in the NPH but associates with faster reduction of pneumococci in the lungs.

### Pre-exposure to Corynebacteria modulates the inflammatory response to *S. pneumoniae* D39

When analyzing the inflammatory mediators in NAL samples from mice pre-treated or not with Corynebacteria and then inoculated with D39 pneumococci, pre-treatment with Corynebacteria associated with higher levels of IL-6 and non-significantly lower levels of TNF in NAL samples compared with samples from mice only inoculated with D39 (**Supplementary Figure 8B** and data not shown).

At 24h post D39 inoculation, BAL samples from mice pre-exposed to Cp1 and/or Cps1 and then inoculated with D39 displayed significantly lower concentrations of TNF, IL-12 and IFNγ as well as trends to lower IL-6 than samples from mice that were only treated with D39 (**Figure 5B**; **Supplementary Figure 8C**). Only MCP-1 displayed higher concentration in BAL samples from mice pre-exposed to Corynebacteria and then inoculated with D39, although not significantly so (**Supplementary Figure 8C**). Thus, in accordance with our *in vitro* results above, pre-exposure to Corynebacteria associate with lower levels of inflammatory mediators in response to pneumococci, which may be related to the reduced pneumococcal burden *in vivo*.

Next, we analyzed the immune cell composition in mice pre-exposed or not to Corynebacteria and then inoculated with D39 pneumococci. NAL samples generally had too few cells to analyze and were excluded from further analyses. Furthermore, apart from an enrichment of CD64^+^Ly6G^-^ macrophages in NPH samples from mice pre-exposed to Cps1 and then inoculated with D39 at 48h post D39 inoculation, only minor changes were observed in NPH samples (**Supplementary Figure 8D**).

At 48h post D39 inoculation, BAL samples from mice pre-exposed to Corynebacteria displayed non-significant trends towards lower levels of CD3^+^CD19^-^ T-lymphocytes and accompanying higher levels of Ly6G^+^CD64^-^ neutrophils compared to mice only inoculated with D39 (**Figures 5C–E**). The levels of CD19^+^CD3^-^ B-lymphocytes, CD64^+^Ly6G^-^ macrophages and CD11c^+^CD64^-^ dendritic cells were largely unaffected (**Figures 5D–F**; **Supplementary Figure 8E**). In lung tissue samples, however, D39 exposed mice pre-treated with Corynebacteria had a significant enrichment of CD3^+^ T-lymphocytes (Cps1), CD19^+^CD3^-^ B-lymphocytes (Cp1), CD64^+^Ly6G^-^ macrophages (Cps1), and CD11c^+^CD64^-^ dendritic cells (Cp1) compared to mice inoculated with only D39 (**Figures 5C–F**; **Supplementary Figure 8E**). The levels of Ly6G^+^CD64^-^ neutrophils were generally comparable to those in lungs from mice only inoculated with D39 (**Figure 5E**).

Combined, these results indicate that the Corynebacteria-associated decrease in pneumococcal burden is linked to a modulated inflammatory response, primarily in the lower RT, compared to pneumococcal treatment alone.

### Intranasal priming with Corynebacteria decrease the pneumococcal burden in the lungs

**Figure 6:**
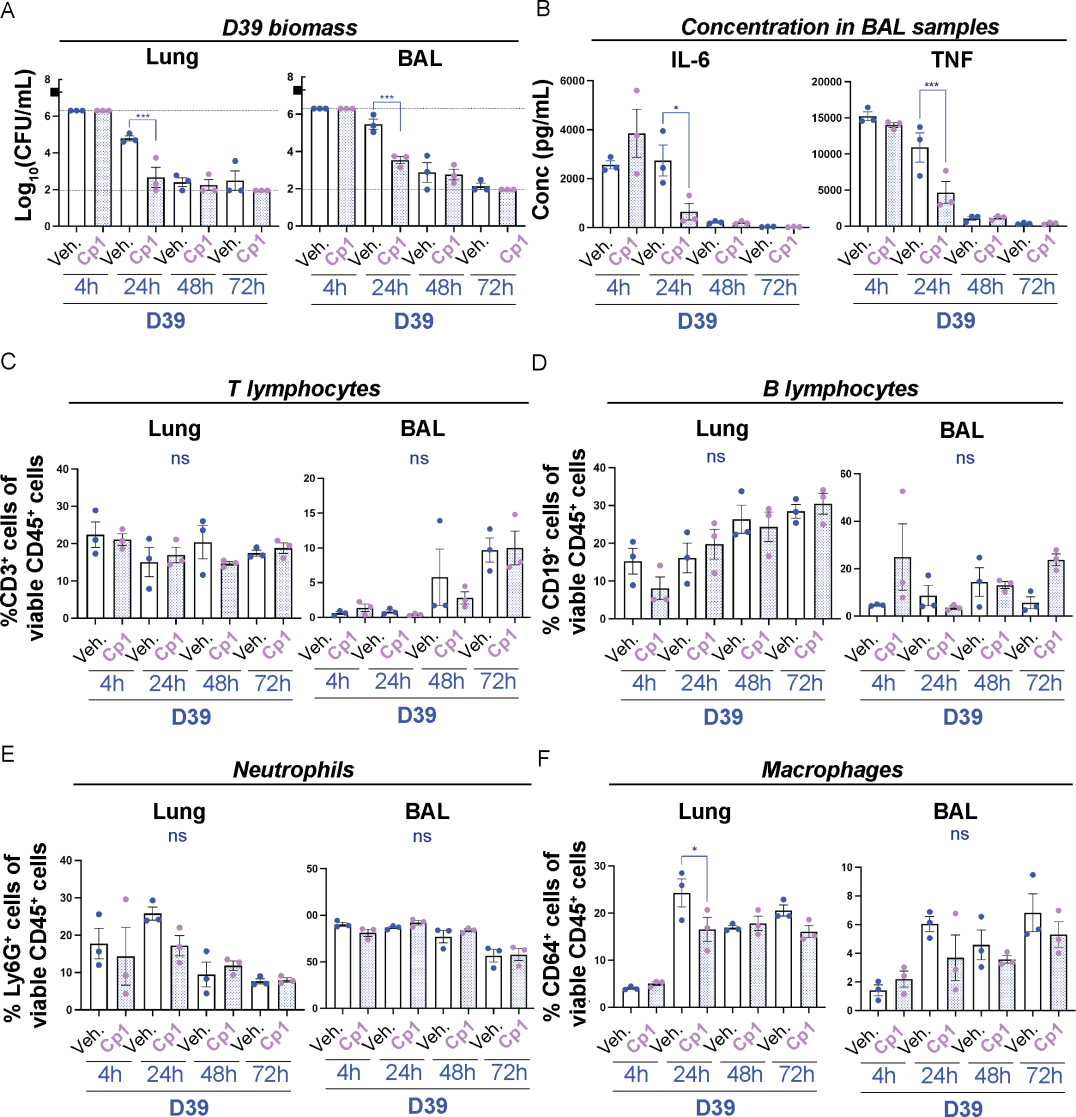
Intranasal priming with Corynebacteria is sufficient to decrease pneumococcal burden and to modulate the immune response in the lungs. Corynebacteria (*C. propinquum* Cp1) were intranasally administered on five consecutive days to BALB/cByJ mice at 10^8^ CFU/mouse/day to allow intranasal colonization. Intranasal administration of PBS served as controls (vehicle; veh.). 24h after the last inoculation, the mice were lightly anesthetized to allow aspiration of 10^7^ CFU/ml D39 pneumococci (indicated by the black box in A). Lung tissue (lung) and bronchoalveolar lavage (BAL) samples were collected 4, 24, 48 or 72h after inoculation with D39. Additional information regarding nasopharynx tissue (NPH), nasal lavage (NAL) and BAL samples is presented in **Supplementary Figure 9**. **(A)** The bacterial burden of D39 pneumococci was determined by viable plate count. Values above and below the detection limits (6.3 and 1.95 log_10_ CFU/mL, respectively) were put to the detection limit (dotted lines). The biomass of Corynebacteria was generally close to or below the detection limit (2-3 log_10_ CFU/mL) in all samples investigated and not shown. **(B)** The concentration of IL-6 (*left panel*) and TNF (*right panel*) in cell-free BAL samples was determined by cytokine bead array (CBA). Values below the detection limit of 5 pg/mL and 7.3 pg/mL for IL-6 and TNF, respectively, were put to the detection limit (dotted lines). **(C–F)**. The percentage of the respective immune cell population was determined by flow cytometry in homogenized lung tissue or pelleted BAL samples with > 4000 viable cells per sample as determined by trypan blue exclusion assay: CD3^+^CD19^-^ T lymphocytes of all viable CD45^+^ cells **(C)**, CD19^+^CD3^-^ B lymphocytes of all viable CD45^+^ cells **(D)**, Ly6G^+^CD64^-^ neutrophils of all viable CD45^+^ cells **(E)**, CD64^+^Ly6G^-^ macrophages of all viable CD45^+^ cells **(F)**. Individual values (mice) with mean and SEM shown from n=3 mice, N=1 experiment. All statistics by ordinary one-way ANOVA with Sidak’s multiple comparison test to D39 only without Corynebacteria priming (blue asterisks over blue lines). ns, not significant, * *P*< 0.05 and *** *P* < 0.001.

To closer mimic nasopharyngeal carriage of Corynebacteria, we next performed intranasal priming that, unlike the aspiration model used above, should result in negligible amount of Corynebacteria in the lungs of inoculated mice at all time points. *C. propinquum* (Cp1) was inoculated in the nares of non-anesthetized mice on five consecutive days to establish colonization in the NPH, as was previously shown for *C. pseudodiphtheriticum* strain 090104 (Kanmani et al., 2017; Ortiz Moyano et al., 2020). PBS-inoculation (vehicle) was used as a control. 24h after the last inoculation, mice were either left untreated or were lightly anesthetized to allow aspiration of PBS or D39 pneumococci. NPH, NAL, lung as well as BAL samples were collected and analyzed over time. Despite the repeated exposure, Corynebacteria were largely undetectable in all niches and at all time points (<2 log_10_ CFU/ml or mouse). The D39 biomass was gradually reduced over time in all niches regardless of pre-treatment (**Figure 6A**; **Supplementary Figure 9A**). The reduction of D39 was, however, significantly faster in lung, BAL and NAL samples from mice intranasally primed with Corynebacteria compared to mice inoculated with D39 alone (**Figure 6A**; **Supplementary Figure 9A**). Interestingly, the observed effects with intranasal priming with Corynebacteria (**Figure 6A**; **Supplementary Figure 9A**) were overall comparable to that for pre-exposure of Corynebacteria by aspiration above (**Figure 5A**; **Supplementary Figure 8A**). This indicates that intranasal priming with Corynebacteria is sufficient to result in a faster reduction of pneumococci also in the lungs.

### Intranasal priming with Corynebacteria modulates the inflammatory response to pneumococci in the lower respiratory tract

To determine whether intranasal priming with Corynebacteria would suffice to modulate the inflammatory response to pneumococci, we next analyzed the inflammatory mediators in NAL and BAL samples. IL-10 was below the detection limit for both NAL and BAL samples and were hence omitted. As observed above, only minor changes in the concentration of inflammatory mediators were observed in NAL samples (**Supplementary Figure 9B** and data not shown). However, in BAL samples, the levels of IL-6, TNF and MCP-1 were significantly lower at 24h in mice intranasally primed with Corynebacteria compared to mice that were only inoculated with D39 pneumococci (**Figure 6B**; **Supplementary Figure 9C**). In contrast, and contrary to the results with pre-exposure by aspiration above (**Supplementary Figure 8C**), the concentration of IFNγ was significantly higher at 24h in BAL samples from primed mice compared to mice inoculated with D39 alone (**Supplementary Figure 9C**). These results indicate that intranasal priming with Corynebacteria is sufficient to modulate pneumococcal-induced release of inflammatory mediators in the lungs.

Finally, we analyzed the immune cell composition in samples from Corynebacteria-primed mice inoculated with D39 pneumococci or PBS (vehicle). Similar to our experiments above, only minor changes were observed in the analyzed immune cell populations in NPH samples (**Supplementary Figure 9D**). A significant decrease in the percentage of CD3^+^CD19^-^ T cells was, however, observed in NPH from mice primed with Corynebacteria and then inoculated with D39 pneumococci at 72h post D39-inoculation (**Supplementary Figure 9D**). In contrast, the levels of CD3^+^CD19^-^ T-lymphocytes increased over time in BAL samples, but were not significantly different between Corynebacteria-primed mice and mice only exposed to D39 in either lung or BAL samples (**Figure 6C**). No significant differences were observed for CD19^+^CD3^-^ B-lymphocytes or Ly6G^+^CD64^-^ neutrophils in either lung or BAL samples (**Figures 6D, E**). However, the level of CD64^+^Ly6G^-^ macrophages were significantly lower in lung samples from Corynebacteria-primed mice compared to mice only exposed to D39 at 24h post D39 inoculation (**Figure 6F**). Similar trends were observed for BAL samples (**Figure 6F**).

Combined, these results indicate that intranasal priming with *C. propinquum* is sufficient to result in a more rapid reduction in pneumococcal burden and associated modulation of the inflammatory response to pneumococci in the lower RT.

## Discussion

Several lines of evidence indicate that Corynebacteria are “keystone species” with strong association to respiratory health (Man et al., 2017). How Corynebacteria interact with host tissues and potential respiratory pathogens during health and disease is, however, largely uncharacterized. In this study, we investigated how Corynebacteria interact with the upper and lower RT tissues alone or when co-inoculated with pneumococci and their modulating activities on pneumococcal burden and host inflammation.

Corynebacteria alone were well tolerated by both human respiratory epithelial cells *in vitro* and the murine RT *in vivo*. Attempts to stably colonize the upper and lower RT of mice with Corynebacteria were unsuccessful as the bacteria were cleared over time in both the NPH (48h) and lungs (24h). Consecutive intranasal inoculation over five days did not improve the colonization density or duration in the RT in our hands, providing us with transient colonization models. To our knowledge, no other study has investigated the biomass of *C. propinquum* and *C. pseudodiphtheriticum* during colonization of the mouse RT. Still, the biomass in the NPH in this study were similar to that reported for *C. accolens* and *C. amycolatum* (Horn et al., 2021), although time points (24h and 48h post inoculation, respectively) and specific experimental setups differ between the studies. The short duration and low density of colonization of the murine RT observed here suggests that Corynebacteria may colonize the human RT better than the mouse RT or that the strains colonizing humans and mouse may be different. This is supported by studies investigating 16S rRNA sequencing from the mouse RT that have detected low levels (Krone et al., 2014; Singh et al., 2017) or no Corynebacteria (Scheiermann and Klinman, 2017; Kostric et al., 2018) during the development of the mouse microbiota, indicating that Corynebacteria is not a major component of the mouse microbiota.

Corynebacteria alone significantly affected the immune landscape in the RT, in a strain- and niche-dependent manner. A transient release of inflammatory mediators was observed *in vitro* and *in vivo*. Interestingly, the anti-inflammatory mediator IL-10 was preferentially induced by *C. propinquum* in BAL samples, indicating species-specific immunomodulatory potential of Corynebacteria. This was accompanied by changes in the immune cell composition that was more pronounced in the lower compared to the upper RT *in vivo*. The effect persisted even after the Corynebacteria were undetectable in the samples. Thus, despite being cleared, exposure to Corynebacteria leaves an imprint on the immune landscape in the RT.

Epidemiological studies have indicated that high abundance of *C. propinquum* and *C. pseudodiphtheriticum* associate with reduced incidence of RTIs in infants (Pettigrew et al., 2012; Biesbroek et al., 2014). In our hands, pre-exposure to or intranasal priming with Corynebacteria did not affect the acquisition of pneumococci in the NPH as was previously observed by Horn et al (Horn et al., 2021). However, in line with the epidemiological data, as well as with previous studies (Horn et al., 2021), we found that the pre-exposure to Corynebacteria was associated with a more rapid reduction of D39 pneumococci in the RT, especially in the lungs. During both experimental setups, significant changes in the immune landscape were observed in mice pre-exposed to Corynebacteria compared to mice that were inoculated with pneumococci alone, which may be related to a direct effect on the immune response as well as the associated reduced pneumococcal burden. Counterintuitively, most inflammatory mediators were lower in samples from mice pre-exposed to Corynebacteria, indicating that the presence of Corynebacteria reduced the pneumococcus-induced inflammation *in vivo*. This is in line with previous studies showing decreased levels of pro-inflammatory mediators in BAL samples from mice pre-exposed to *C. accolens* or *C amycolatum* and subsequently infected with pneumococci (Horn et al., 2021). On the other hand, the levels of specific immune cell populations were affected in mice pre-exposed to Corynebacteria. The levels of T- and B-lymphocytes as well as macrophages were generally higher, whereas neutrophils tended to decline. One caveat is, however, that the differences in total numbers of these populations were not evaluated. Nevertheless, these results are overall consistent with those reported by Horn et al (Horn et al., 2021). The host’s immune response, rather than the bacteria themselves, causes many of the symptoms and much of the tissue damage during pneumonia. Thus, it is possible that the previously induced low-grade inflammatory response by Corynebacteria in the lungs is protective against severe disease while likely improving clearance of pneumococci. Additionally, a previous study has shown that pneumococcal strains that induce low NFκB activity are more virulent and cause more severe disease in mice compared to strains inducing high NFκB activity (Coleman et al., 2017). Thus, it is possible that the previous activation of NFκB by Corynebacteria may be involved in modulating inflammation following and preventing severe pneumonia after pneumococcal inoculation. This is also in line with the proposal that local, subclinical, inflammation may protect against pneumococcal pneumonia (Drigot and Clark, 2024). It should, however, also be noted that interpretation of our results is limited by the small number of data points (three mice per group) yet warrants further studies with larger sample size.

Previous studies have indicated that specific Corynebacteria strains (e.g., *C. pseudodiphtheriticum* strain 090104) may protect mice against pneumococcal and RSV-induced pneumonia (Kanmani et al., 2017; Ortiz Moyano et al., 2020). Apart from *C. pseudodiphtheriticum*, we also show here that *C. propinquum* may protect against pneumococcal pneumonia. *In vitro* studies have indicated that *Corynebacterium* spp. inhibit the growth of pneumococci by release of fatty acids or unknown mechanisms (Bomar et al., 2016; Xu et al., 2021). In our hands, neither supernatants from Corynebacteria, nor the presence of live Corynebacteria, had any significant effect on the viability or growth of D39 pneumococci. Additionally, in our *in vivo* studies, Corynebacteria were cleared from the RT over time and was largely undetectable at the time of pneumococcal inoculation. Thus, although we cannot fully rule out a direct competitive effect *in vivo*, the Corynebacteria-associated reduction in pneumococcal biomass is most likely mediated by the modulated inflammatory response described above. The specific mechanisms involved will be explored further in future studies.

To summarize, we show that clinical isolates of the RT commensal Corynebacteria are well tolerated and induce a niche-associated and transient inflammatory response *in vitro* and *in vivo*. Pre-exposure to or intranasal priming with Corynebacteria associate with a faster reduction of pneumococci in the RT, which is likely mediated by modulation of the inflammatory response to pneumococci.

## Data Availability

The original contributions presented in the study are included in the article/**Supplementary Material**. Further inquiries can be directed to the corresponding author.
