# Supplementary figures and images for: Corynebacteria from the respiratory microbiota modulate inflammatory responses and associate with a reduced pneumococcal burden in the lungs
Source: Front Cell Infect Microbiol. 2025 Jan 28;14:1530178. doi: 10.3389/fcimb.2024.1530178 (PMC11811110; doi:10.3389/fcimb.2024.1530178)

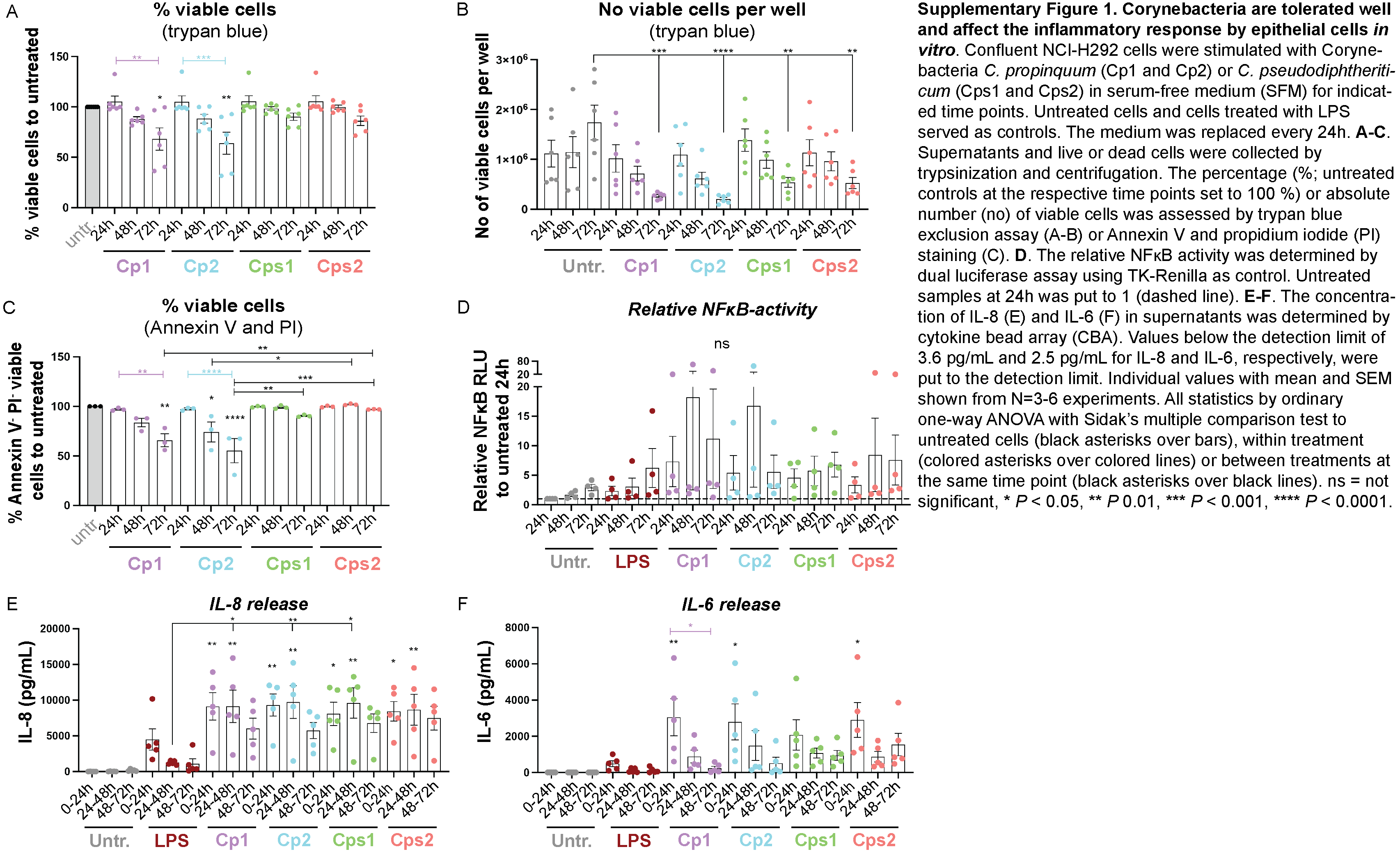

Supplement: Supplementary file 1 [file Image1.tif]

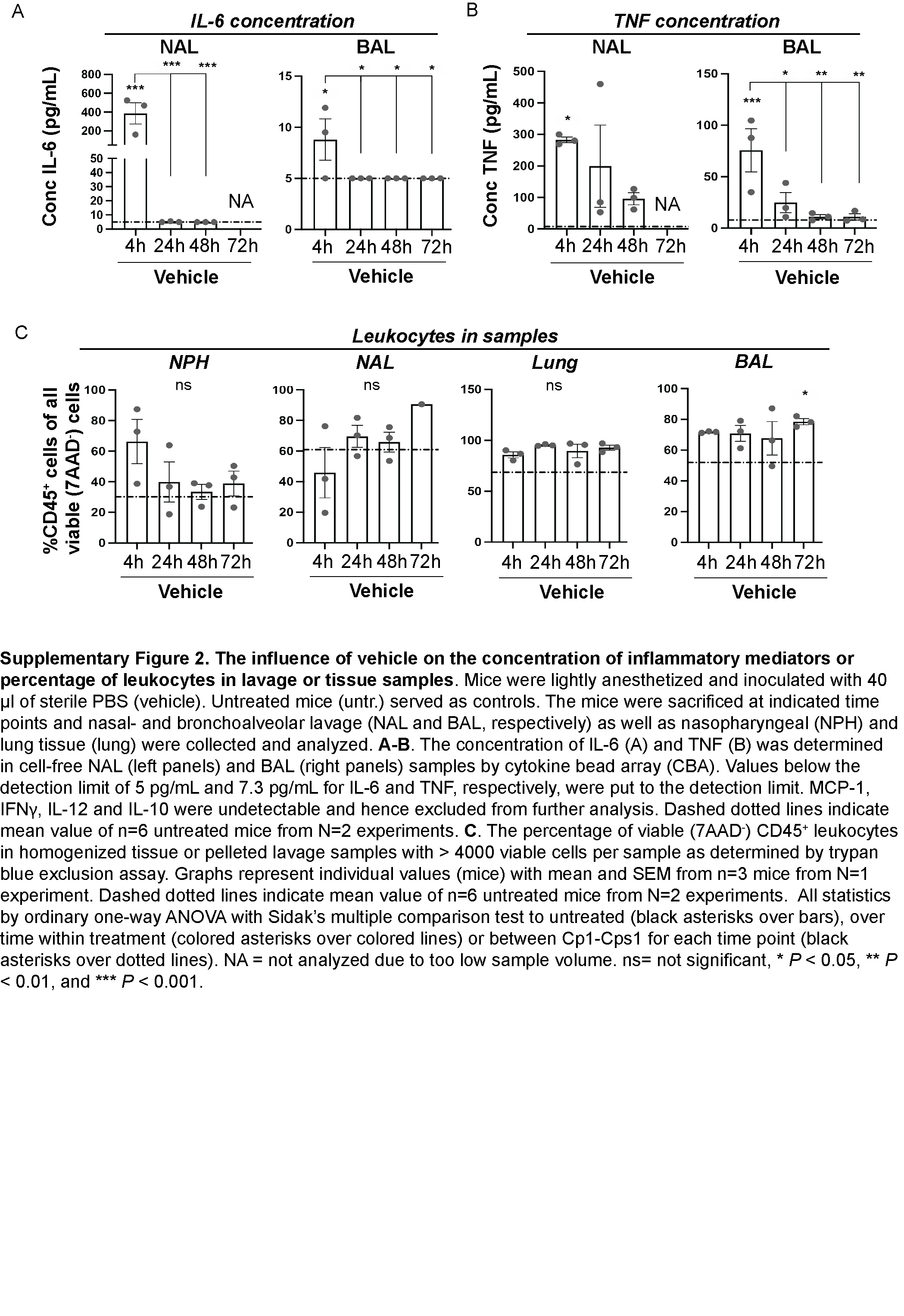

Supplement: Supplementary file 2 [file Image2.tif]

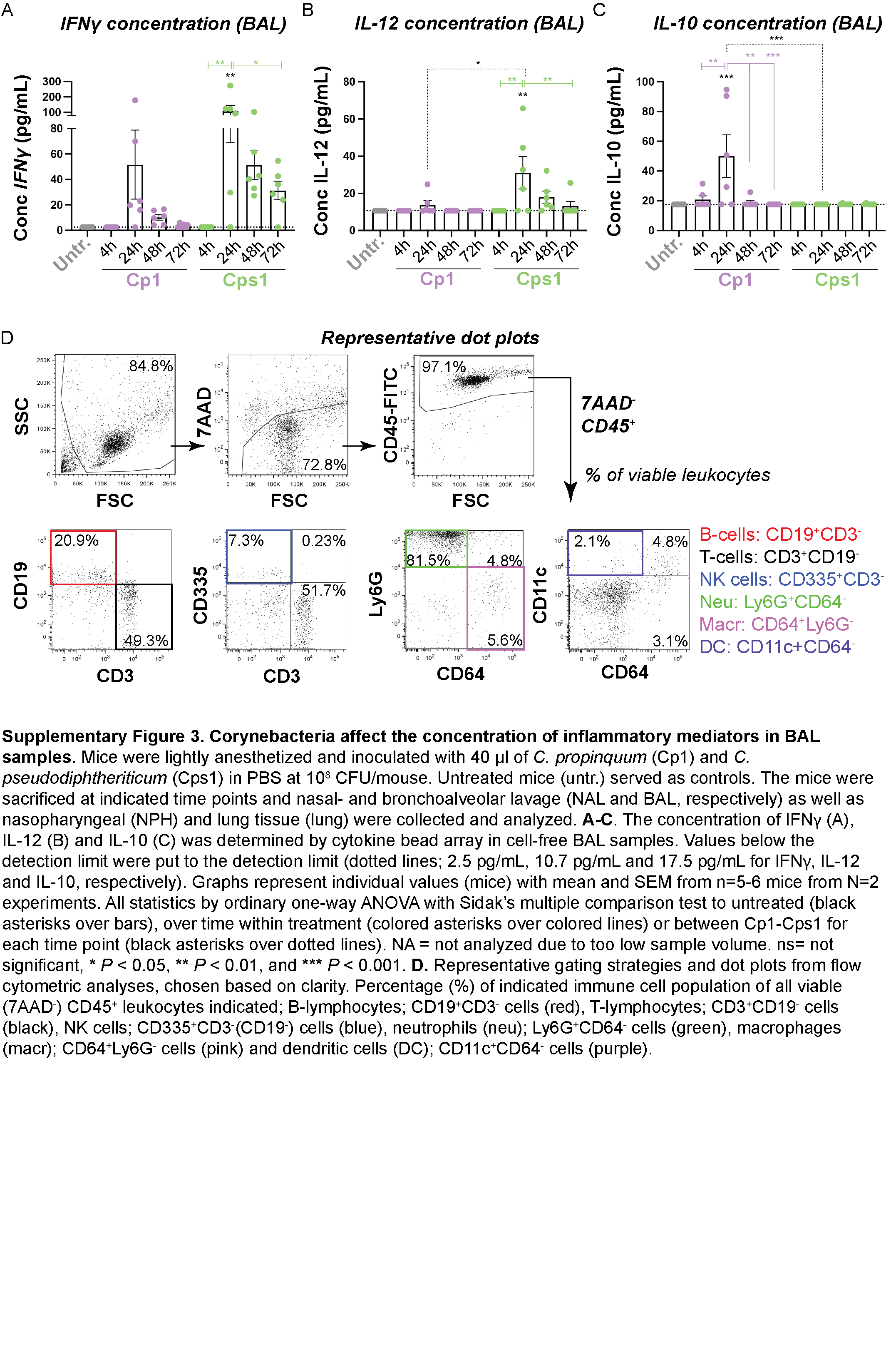

Supplement: Supplementary file 3 [file Image3.tif]

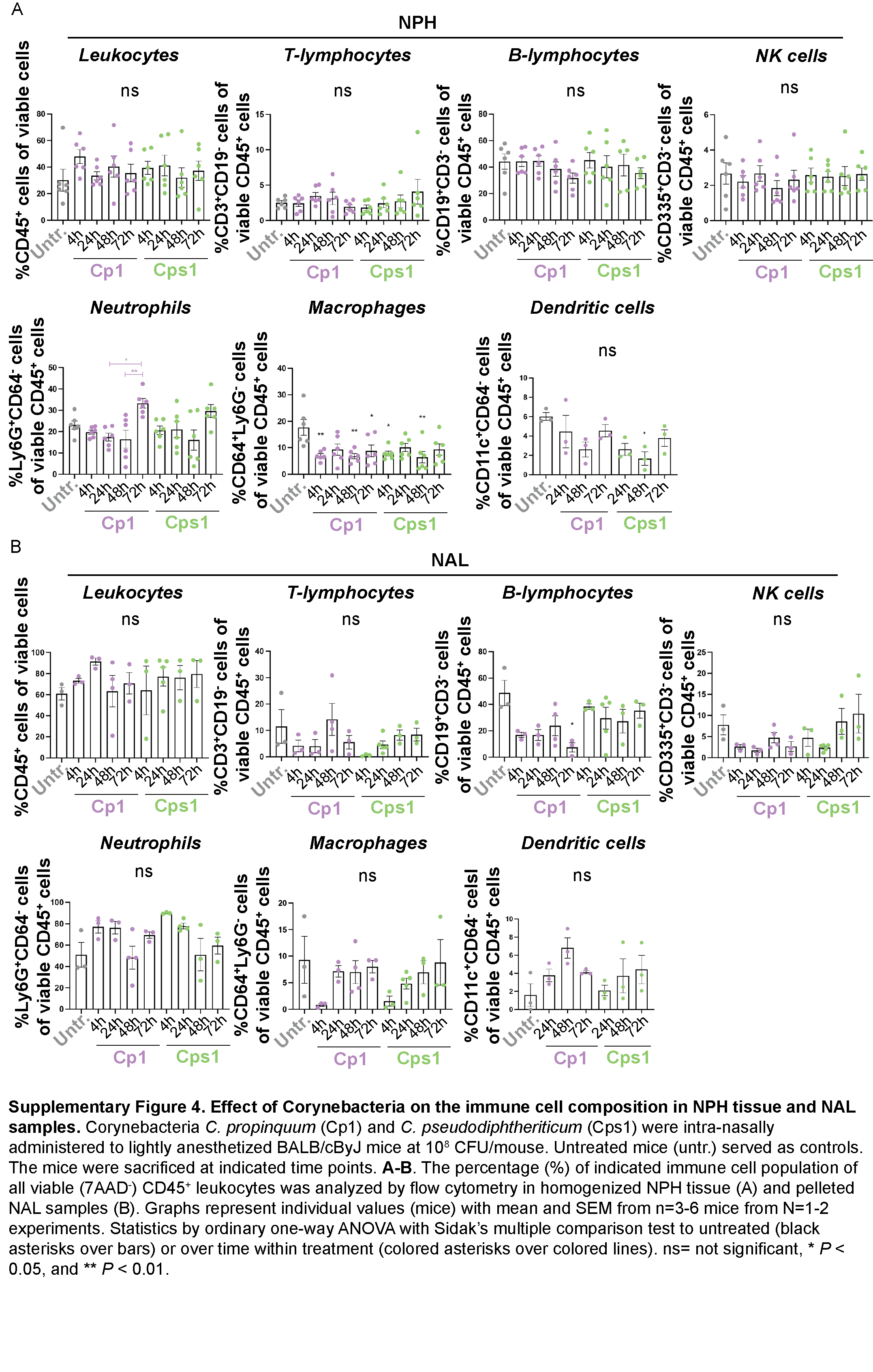

Supplement: Supplementary file 4 [file Image4.tif]

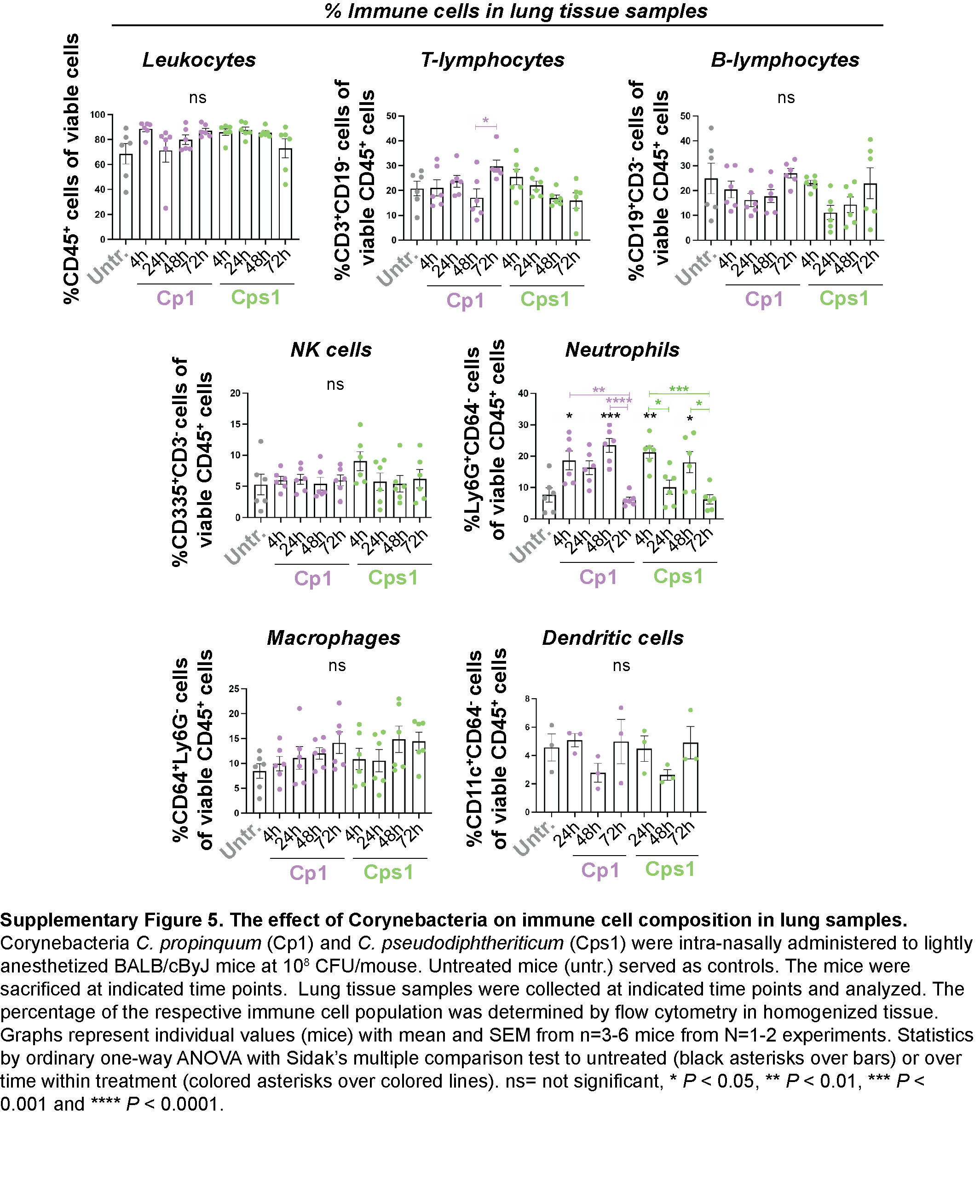

Supplement: Supplementary file 5 [file Image5.tif]

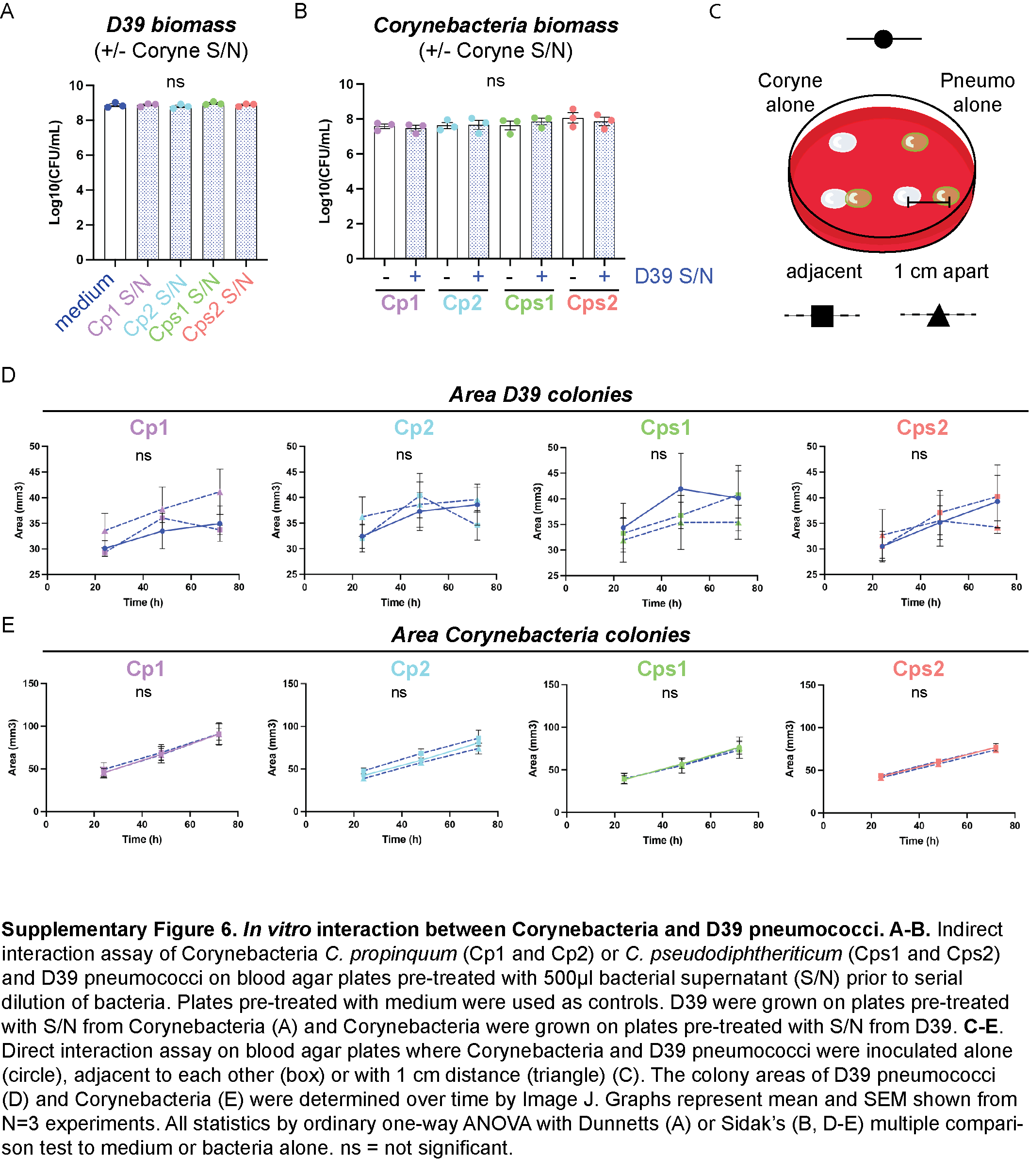

Supplement: Supplementary file 6 [file Image6.tif]

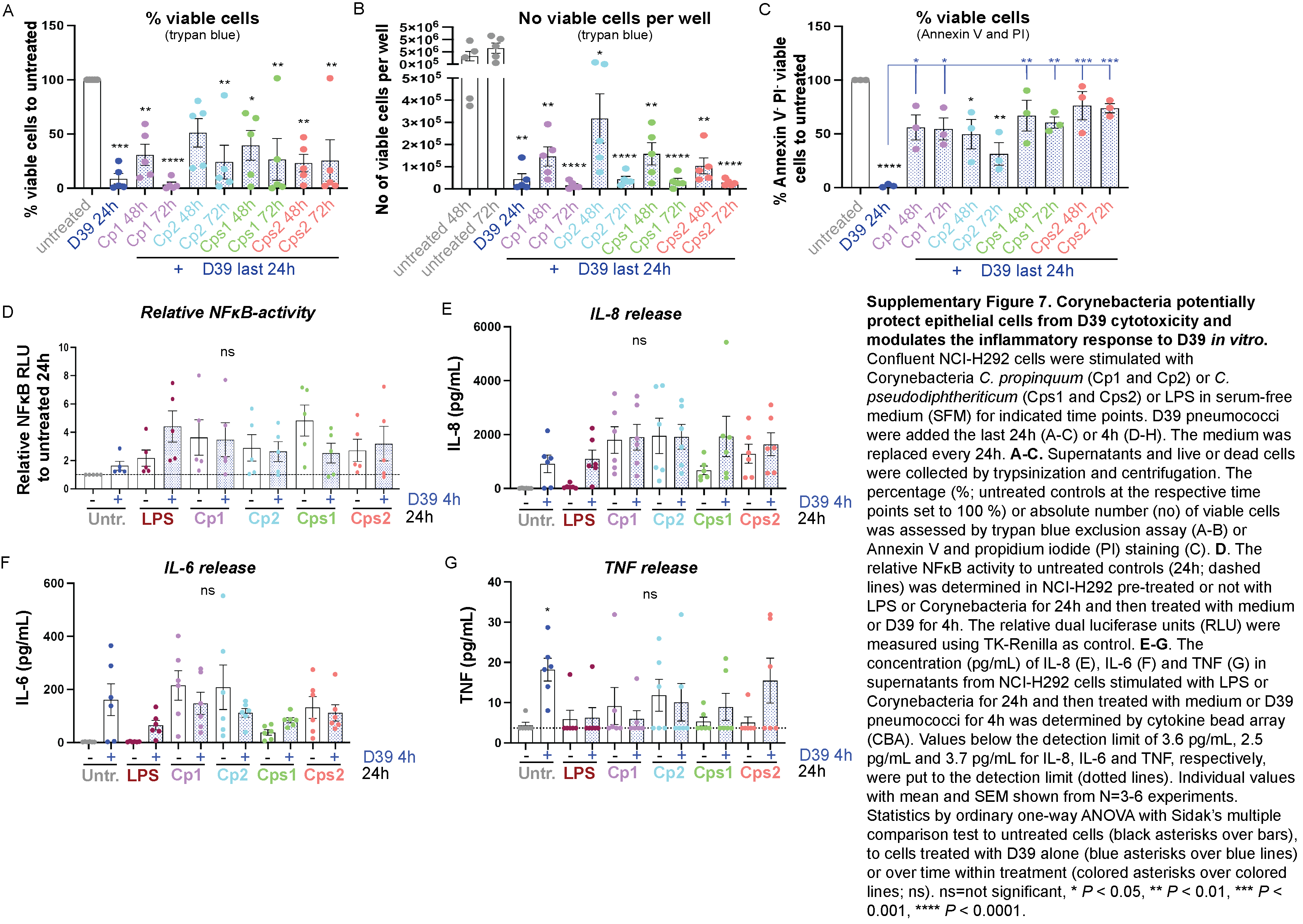

Supplement: Supplementary file 7 [file Image7.tif]

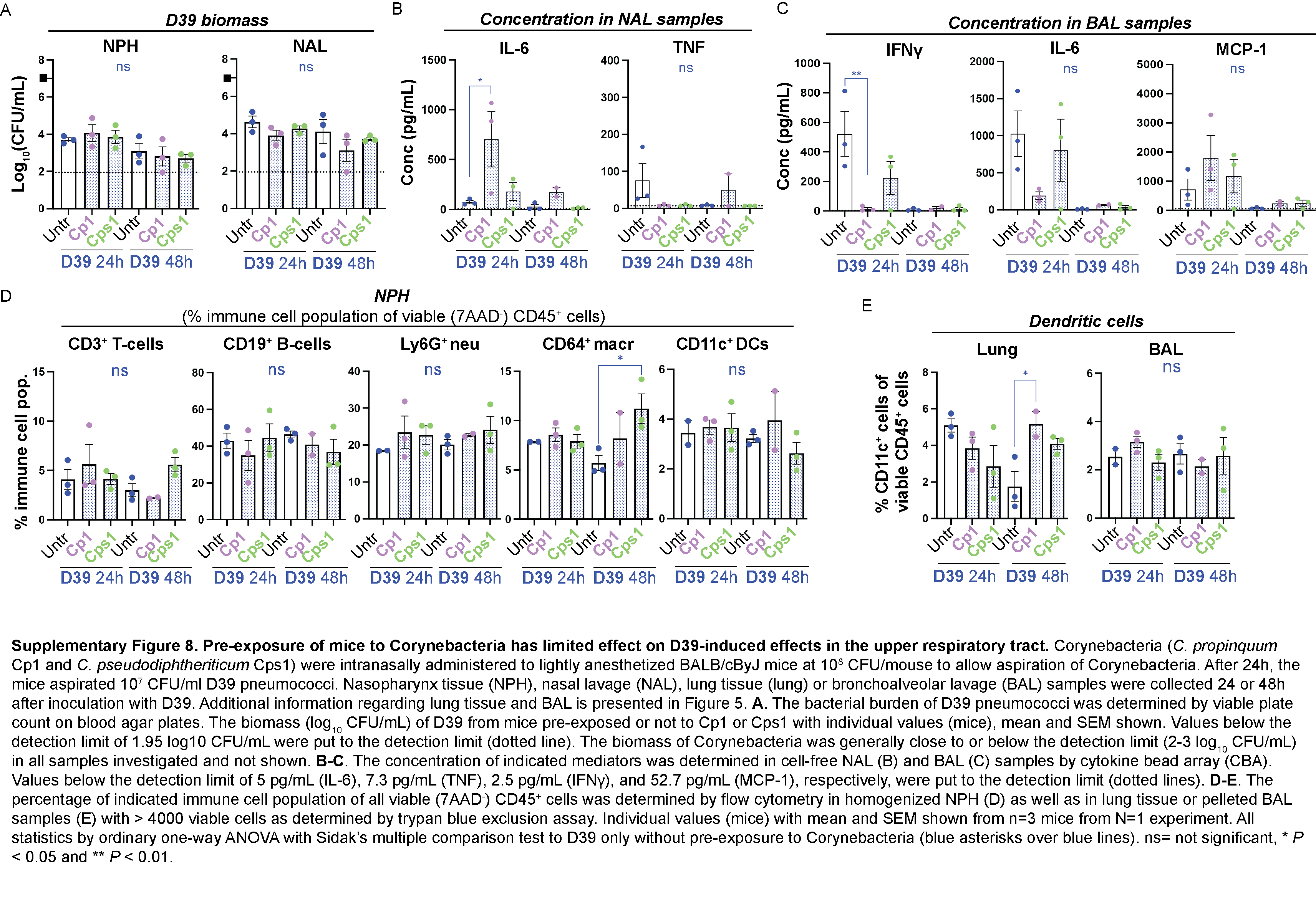

Supplement: Supplementary file 8 [file Image8.tif]

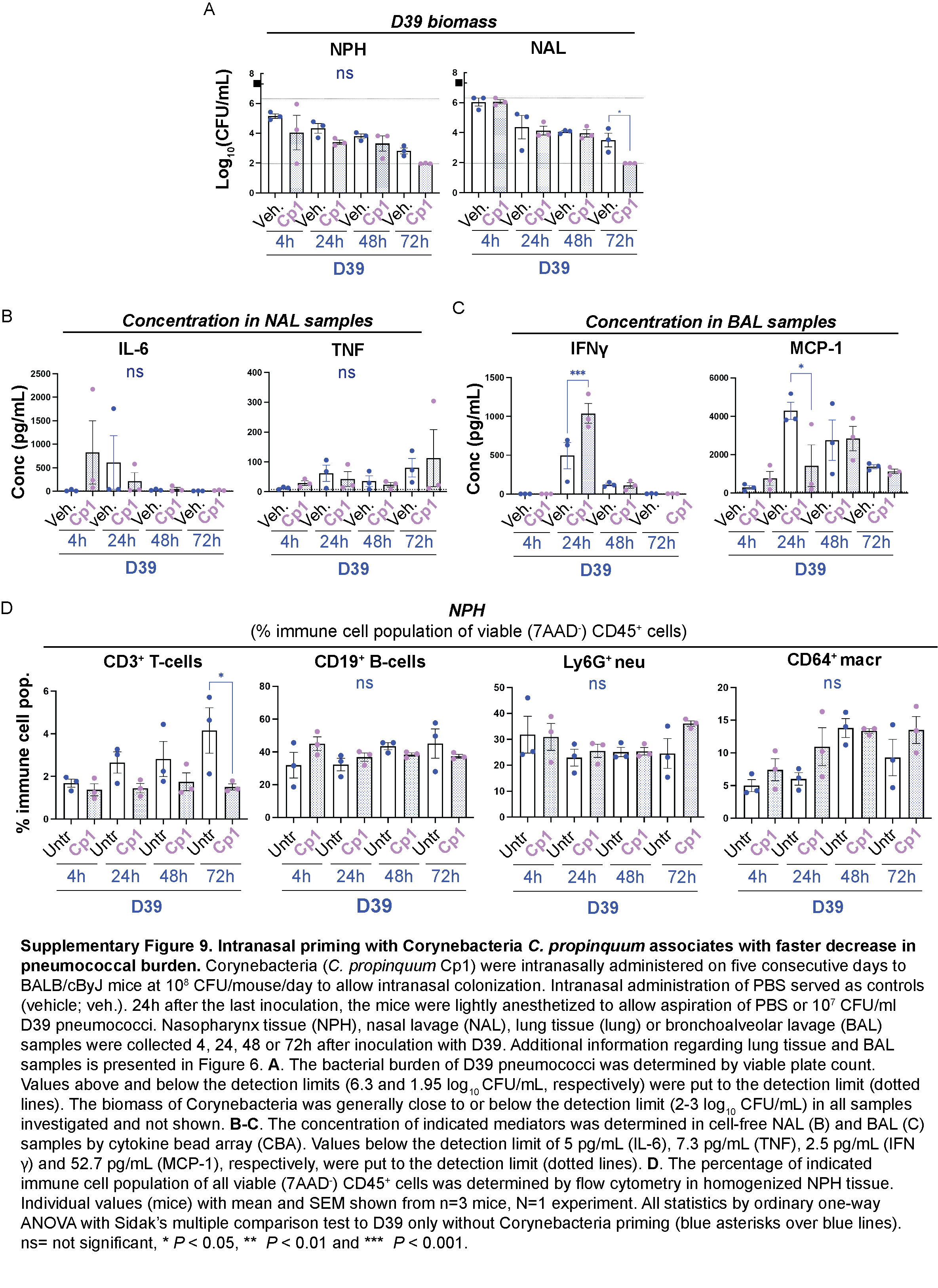

Supplement: Supplementary file 9 [file Image9.tif]
